# Single-Use vs Reusable Catheters for Intermittent Catheterization in Patients With Urinary Retention: The COMPARE Randomized Clinical Trial

**DOI:** 10.1001/jamanetworkopen.2026.20871

**Published:** 2026-06-30

**Authors:** Felice E. E. van Veen, Coen H. H. Christiaans, Sophie A. Berendsen, Tess van Doorn, Jessica L. Boekhorst, Selma Koedood, Maartje Kuenen, Anita W. T. M. Roelofs, Lambertus P. W. Witte, Siebren Dijkstra, Jos van Brakel, Gerard J. van der Wielen, Luc A. J. Roelofs, Josien H. Wolterbeek, Evert L. Koldewijn, Joost van Asten, Ivar Bleumer, Bettina E. Hansen, Jeroen R. Scheepe, Bertil F. M. Blok

**Affiliations:** 1 Department of Urology, Erasmus University Medical Center, Rotterdam, the Netherlands; 2 Department of Urology, Zuyderland Medical Center, Heerlen, the Netherlands; 3 Department of Urology, Rijnstate, Arnhem, the Netherlands; 4 Department of Urology, Isala, Zwolle, the Netherlands; 5 Department of Urology, Canisius-Wilhelmina Hospital, Nijmegen, the Netherlands; 6 Department of Urology, Amphia Hospital, Breda, the Netherlands; 7 Department of Urology, Haaglanden Medical Center, the Hague, the Netherlands; 8 Department of Urology, Treant Zorggroep, Emmen, the Netherlands; 9 Department of Urology, Franciscus Gasthuis and Vlietland, Rotterdam, the Netherlands; 10 Department of Urology, Catharina Hospital, Eindhoven, the Netherlands; 11 Department of Urology, HagaZiekenhuis, the Hague, the Netherlands; 12 Department of Urology, Sint Jansdal, Harderwijk, the Netherlands; 13 Department of Epidemiology and Biostatistics, Erasmus University Medical Center, Rotterdam, the Netherlands

## Abstract

**Importance:**

Clean intermittent catheterization (CIC) is the preferred management for urinary retention. Single-use catheters generate substantial environmental and financial burdens, whereas evidence on the safety of reusable catheters is limited.

**Objective:**

To examine whether reusable catheters are noninferior to single-use catheters for CIC with respect to urinary tract infection (UTI) incidence.

**Design, Setting, and Participants:**

This multicenter, noninferiority randomized clinical trial was conducted between February 21, 2020, and April 3, 2025, at 12 Dutch hospitals and included patients aged 16 years or older performing CIC at least twice daily, with 1-year follow-up.

**Interventions:**

Participants were randomly assigned to reusable or single-use catheters. Reusable catheters were disinfected daily in 2% sodium hypochlorite solution, rinsed before and after use, and replaced every 2 weeks; single-use catheters were permitted for 20% or less of weekly catheterizations in the reusable group.

**Main Outcomes and Measures:**

The primary outcome was UTI incidence per patient-month. Secondary outcomes included catheter-related complications, adverse events, and patient-reported outcomes. The noninferiority margin was set at an absolute difference of 0.07 UTIs per patient-month.

**Results:**

A total of 386 patients (mean [SD] age, 61.4 [15.9] years; 243 [63%] male; and 139 [36%] with neurogenic lower urinary tract dysfunction) were included. In the reusable catheter group, 72 of 185 participants (39.0%) discontinued study participation, mainly due to reduced ease of use and urethral irritation. In the modified intention-to-treat population (n = 326), mean (SD) follow-up was 12.0 (1.9) months in the single-use group and 11.4 (2.9) months in the reusable group. During this period, 1 or more UTIs occurred in 59 of 191 single-use catheter users (30.9%) and 40 of 134 reusable catheter users (29.9%). The incidence rate was 0.054 (95% CI, 0.040-0.069) vs 0.050 (95% CI, 0.035-0.067) UTIs per patient-month, with an absolute difference of −0.004 (95% CI, −0.025 to 0.019), meeting the predefined noninferiority margin. Catheter-related complications and serious adverse events were comparable between groups, whereas low-grade adverse events, mainly urethral irritation, were more frequent in the reusable group (37 [20.0%] vs 9 [4.7%]). Catheter-related quality of life was higher with reusable catheters, whereas catheterization satisfaction was higher with single-use catheters; overall health-related quality of life did not differ between groups.

**Conclusions and Relevance:**

In this randomized clinical trial of patients with urinary retention, reusable catheters were noninferior to single-use catheters for UTIs, with variable tolerability and patient-reported outcomes highlighting the importance of individual preference and the need for design enhancements to improve usability and broaden implementation.

**Trial Registration:**

National Trial Register: NL8296

## Key Points

**Question:**

Are reusable catheters noninferior to single-use catheters for intermittent catheterization in cases of urinary tract infections?

**Findings:**

In this randomized clinical trial of 386 participants, the incidence rate of urinary tract infections per patient-month was similar between the single-use and reusable catheter groups, and the predefined noninferiority margin was met.

**Meaning:**

This study’s findings suggest that reusable catheters are as safe as single-use catheters, thereby supporting sustainable and equitable urologic care.

## Teaser

This randomized clinical trial compares urinary tract infection incidence in patients aged 16 years or older using single-use vs reusable catheters in Dutch hospitals.

## Introduction

Urinary retention affects millions of people worldwide,^1^ with clean intermittent catheterization (CIC) being the preferred treatment.^2,3^ Patients typically perform CIC 4 to 6 times daily, maintaining catheterized volume below 400 to 500 mL. Catheters used for CIC are either single use or reusable. Single-use catheters are discarded after each use and often prelubricated or coated for ease of insertion, whereas reusable catheters are uncoated devices cleaned and reused multiple times. The widespread use of single-use catheters generates substantial environmental and financial burdens, producing an estimated 206 million L of nonbiodegradable waste annually in the US alone and costing more than US $3 billion per year.^4,5^

Reusable catheters offer a potentially sustainable alternative, but concerns about urinary tract infection (UTI) risk have limited their use. Evidence comparing single-use and reusable catheters is limited and of low certainty. Small randomized clinical trials, including the largest with 80 participants, and systematic reviews^6,7^ have not demonstrated clear differences in UTI rates, and adverse events (AEs) and patient-reported outcomes (PROs) have been infrequently reported.

To address this gap, we conducted the Single Use vs Reusable Catheters in Intermittent Catheterization for Treatment of Urinary Retention (COMPARE) trial^8^ to assess whether reusable catheters are noninferior to single-use catheters in terms of UTI incidence. Secondary objectives included evaluating safety outcomes (ie, catheter-related complications and AEs), efficiency (ie, patient satisfaction and quality of life), and cost-effectiveness. This analysis focuses on the primary, safety, and efficiency outcomes.

## Methods

### Study Design

This multicenter, noninferiority randomized clinical trial was conducted between February 21, 2020, and April 3, 2025, at 12 hospitals in the Netherlands, approved by the institutional review board of the Erasmus University Medical Center, and prospectively registered in the National Trial Register. Patients were recruited from outpatient urology clinics. Written informed consent was obtained before enrollment. Patients were followed up for 1 year, including 9 follow-up visits. The first 6 weeks were considered a lead-in phase, during which patients adjusted to using the reusable catheter. During each visit, UTIs, catheter-related complications, AEs, and treatment adherence were assessed. Safety and study adherence were monitored by an independent data and safety monitoring board. This study followed the Consolidation Standards of Reporting Trials (CONSORT) guideline. Detailed study methods have been published previously,^8^ and the full trial protocol and statistical analysis plan are available in Supplement 1.

### Participants

Eligible patients were 16 years or older with urinary retention due to neurogenic lower urinary tract dysfunction (NLUTD) or non-NLUTD, who had been performing CIC 2 times or more daily for at least 2 weeks. Exclusion criteria included temporary catheterization (<12 months), nonurethral catheterization, urethral strictures, urinary tract stones, bladder augmentation, active bladder cancer follow-up, immunosuppressive use, or neurocognitive impairment precluding study comprehension.

### Intervention and Randomization

Participants were randomly assigned to either reusable catheters (Cliny [Create Medic Co Ltd] for males and PureCath [QA Medical Co Ltd] for females) or continued use of their single-use catheters. Reusable uncoated catheters were used for 2 weeks. Additional lubricant was provided if needed. The catheters were rinsed with tap water before and after each use and stored in a 2% sodium hypochlorite solution (1:80 dilution) with cold tap water, renewed daily.^9^ To enhance adherence, participants were allowed to use single-use catheters for up to 20% of weekly catheterizations.

Randomization used computerized minimization, stratified by study site, sex, age (16-17, 18-49, and ≥50 years), catheterization cause (NLUTD vs non-NLUTD), and menopausal status (for females). Data on race were collected to describe the characteristics of the study population. Race was investigator-reported based on participant-reported ancestry and/or ethnic background (African, Asian, Latin American, White, or unknown). No racial categories were collapsed for analysis. Participants and clinical research staff were unblinded due to the visible and procedural differences between catheter types. Outcome assessors were masked to group allocation.

### Study Outcomes

The primary outcome was UTI incidence per patient-month during follow-up. UTIs were defined according to the criteria by Woodford and George,^10^ consistent with guidelines from the European Association of Urology^2^ and Dutch College of General Practitioners.^11^ Diagnosis required the acute onset of 1 or more UTI symptoms and a positive urine test result (culture, sediment, dipslide, or nitrite test), performed either in the hospital or by a general practitioner. UTI symptoms included dysuria, pain during catheterization, urinary frequency or urgency, suprapubic pain, hematuria, flank pain, fever (temperature >38 °C), rigors, or delirium. For patients with NLUTD, increased incontinence, limb spasms, and autonomic dysregulation were also considered indicative. Secondary safety outcomes included UTI-related hospitalizations, bacteremic UTI, systemic infections (eg, pyelonephritis, epididymo-orchitis, and prostatitis), kidney or bladder stones, urethral strictures, and macroscopic hematuria. AEs were recorded according to the Common Terminology Criteria for Adverse Events (CTCAE), version 5.0, with grade 1 indicating mild; grade 2, moderate; grade 3, severe; grade 4, life-threatening; and grade 5, death related to AEs. Serious adverse events (SAEs) were defined as events of grade 3 or higher.

PROs, including catheter-related quality of life (Intermittent Self-Catheterization Questionnaire), catheterization satisfaction (Intermittent Catheterization Satisfaction Questionnaire), and health-related quality of life (EuroQoL 5-dimension 5-level questionnaire and Qualiveen Short Form), were assessed at baseline and at weeks 6, 26, and 52. Cost-effectiveness analyses are reported separately.

### Sample Size Calculation

A total of 386 participants (193 per group) were planned to demonstrate noninferiority of reusable vs single-use catheters for monthly UTI incidence.^6,12^ The noninferiority margin was set at a 0.07 UTI per patient-month absolute difference (based on 50% of the means, following Althunian et al^13^), with 80% power and a 1-sided α = .025.^6,12^ The calculation accounted for an anticipated 20% attrition and 1-year follow-up.

### Statistical Analysis

Analyses were conducted according to a prespecified statistical analysis plan; detailed information is available in Supplement 1. The primary analysis was performed in the modified intention-to-treat (mITT) population, including all randomized patients with 6 weeks or more of follow-up (ie, those who attended the first clinical follow-up visit after the lead-in phase). Sensitivity analyses were conducted in the intention-to-treat (ITT) population (all randomized patients who initiated treatment regardless of protocol adherence) and the per-protocol population (patients who completed the 12-month follow-up without major deviations).

For the primary outcome, UTI incidence per patient-month was calculated with 95% CIs, which were estimated using a percentile bootstrap procedure with 2000 iterations. The absolute difference between groups was calculated and reported with 2-sided bootstrap 95% CIs. Noninferiority was concluded if the upper limit of the CI was below the noninferiority margin of 0.07 UTIs per patient-month. Incidence rate ratios (IRRs) with 95% CIs were estimated using a negative binomial regression model to account for overdispersion. Time to first UTI was analyzed using Kaplan-Meier survival analysis, and groups were compared using the log-rank test.

Missing data on UTIs were minimal (<5% in both ITT and mITT populations); complete case analysis was therefore used as the primary approach. To assess robustness, we performed a worst-case sensitivity analysis, substituting missing values in the reusable catheter arm with the maximum observed UTI incidence rate, with missing values in the single-use arm left unchanged.

To account for nonadherence in the reusable group, a complier average causal effect (CACE) analysis was performed within the mITT population, using randomization as an instrumental variable for actual catheter use. To address potential clustering within hospitals, a sensitivity analysis using cluster-robust SEs was performed.

PROs were analyzed using linear mixed-effects models with treatment group, time, and their interaction as fixed effects and a random intercept per participant to account for within-subject correlation. No covariate adjustment was deemed necessary given the stratified randomization. Analyses were conducted under a missing at random assumption. The primary estimand for PRO comparisons was the treatment difference at week 52.

Dichotomous secondary outcomes were analyzed using χ^2^ tests or Fisher exact tests, and continuous outcomes using independent-samples *t* tests or Mann-Whitney *U* tests. All tests were 2-sided, with *P* < .05 considered statistically significant. Analyses were performed in R, version 4.4.1 (R Foundation for Statistical Computing).

## Results

### Study Population

A total of 386 patients (mean [SD] age, 61.4 [15.9] years; 243 [63.0%] male, 142 [37.0%] female; 3 African [0.8%], 4 Asian [1.0%], 3 Latin American [0.8%], 373 White [96.6%], and 3 unknown race [0.8%]; and 139 [36%] with NLUTD) participated in the study. A total of 248 participants (64.2%) performed CIC more than 5 times per day, and 165 (42.7%) had more than 3 years of CIC experience. Baseline demographic and clinical characteristics are summarized in Table 1.

**Table 1.** Demographics and Baseline Characteristics

Characteristic	No. (%) of participants^a^	
	Single-use catheter (n = 193)	Reusable catheter (n = 193)
Age, mean (SD) [range], y	61.1 (15.3) [20.1-83.9]	61.7 (16.5) [20.4-85.7]
BMI, mean (SD)	25.6 (4.0)	26.1 (4.5)
Sex		
Female	71 (37.0)	71 (37.0)
Male	122 (63.0)	122 (63.0)
Menopausal status^b^		
Premenopausal	23 (32.4)	20 (28.2)
Perimenopausal	5 (7.0)	5 (7.0)
Postmenopausal	43 (60.6)	46 (64.8)
Race		
African	0	3 (1.6)
Asian	1 (0.5)	3 (1.6)
Latin American	2 (1.0)	1 (0.5)
White	189 (98.4)	184 (96.3)
Unknown	1 (0.5)	2 (1.0)
Comorbidities		
Cardiovascular disease	43 (22.3)	37 (19.2)
COPD	9 (4.7)	8 (4.1)
Diabetes	13 (6.7)	16 (8.3)
Hypertension	50 (25.9)	47 (24.4)
NLUTD	69 (36.0)	70 (36.0)
Multiple sclerosis	12 (17.4)	15 (21.4)
Spina bifida	12 (17.4)	12 (17.1)
Spinal cord injury	28 (40.6)	22 (31.4)
Others	24 (24.6)	21 (30.0)
Non-NLUTD	124 (64.0)	123 (64.0)
BPH	11 (5.7)	15 (7.8)
Postoperative	16 (8.3)	12 (6.2)
Underactive bladder	85 (44.1)	84 (43.5)
Others	12 (6.2)	12 (6.2)
Spontaneous miction		
Yes	107 (55.0)	89 (47.0)
No	86 (45.0)	99 (53.0)
Unknown	0	5
Hand function		
Normal	183 (95.8)	174 (92.1)
Mild impairment	7 (3.7)	12 (6.3)
Severe impairment	1 (0.5)	3 (1.6)
Unknown	2	4
CIC experience, y		
<0.5	22 (11.6)	20 (10.4)
0.5-1.0	20 (10.5)	32 (16.7)
1.0-3.0	66 (34.7)	57 (29.7)
>3.0	82 (43.2)	83 (43.2)
Unknown	3	1
CIC frequency, times per day		
2-4	73 (38.0)	59 (31.0)
5-6	81 (43.0)	94 (49.0)
≥7	36 (19.0)	37 (19.0)
Unknown	3	3
Antibiotic prophylaxis		
None	173 (90.1)	171 (90.0)
Nitrofurantoin	16 (8.3)	15 (7.9)
Others	3 (1.6)	4 (2.1)
Unknown	1	3
Bladder irrigation		
None	166 (86.5)	173 (91.1)
Chondroitin sulfate	1 (0.5)	0
Gentamicin	1 (0.5)	2 (1.1)
Water	20 (10.4)	14 (7.4)
Others	4 (2.1)	1 (0.5)
Unknown	1	3
Intradetrusor botulinum toxin A	35 (18.0)	38 (20.0)
Self-reported UTIs in previous 6 mo before trial entry		
0	134 (69.0)	132 (68.0)
1-2	51 (26.0)	48 (25.0)
>3	8 (4.1)	13 (6.7)

Abbreviations: BMI, body mass index (calculated as weight in kilograms divided by the height in meters squared); BPH, benign prostatic hyperplasia; CIC, clean intermittent catheterization; COPD, chronic obstructive pulmonary disease; NLUTD, neurogenic lower urinary tract dysfunction; UTI, urinary tract infection.

^a^ Unless otherwise indicated.

^b^ Only females included (n = 71).

Of the 386 participants, 378 initiated treatment and were included in the ITT population (single-use: 192 of 193 [99.5%]; reusable: 185 of 193 [95.9%]). At week 6, 326 patients had completed the lead-in phase and were included in the mITT population (single-use: 191 of 192 [99.5%]; reusable: 134 of 185 [72.4%]). Between weeks 6 and 52, an additional 36 patients discontinued (single-use: 14; reusable: 22), resulting in 72 of 185 patients (39.0%) discontinuing in the reusable catheter group. Discontinuations were primarily due to urethral irritation (13 of 185 [7.0%]) or ease-of-use challenges (39 of 185 [21.1%]). These included difficulties with insertion due to insufficient catheter stiffness or smoothness (22 of 185 [11.9%]), additional handling steps required for reuse (eg, rising before and after use or changing cleaning solution; 19 of 185 [10.3%]), and a catheter that was too short (5 of 185 [2.7%]); patients could report more than one ease-of-use challenge.

**Figure 1.**
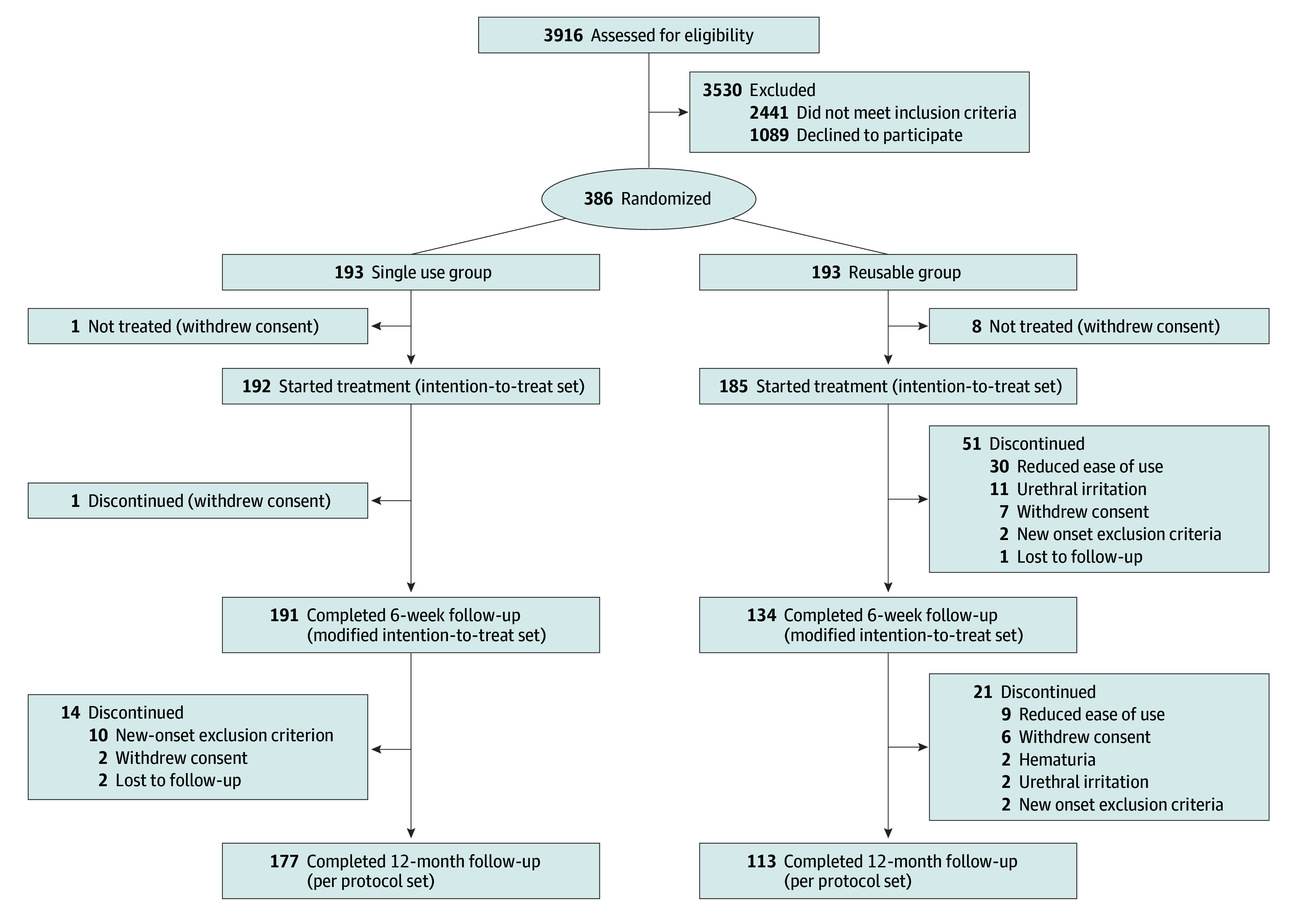
Flowchart for the COMPARE Trial COMPARE indicates the Single Use vs Reusable Catheters in Intermittent Catheterization for Treatment of Urinary Retention trial.

A total of 290 patients completed the 12-month follow-up and were included in the per-protocol population (single-use: 177 of 191 [92.7%]; reusable: 113 of 134 [84.3%]) (Figure 1). No significant differences in baseline characteristics were observed among the ITT, mITT, and per-protocol populations (eTable 1 in Supplement 2). To explore potential dropout bias, we performed logistic regression analyses within the reusable catheter group. The only significant baseline factor associated with dropout was prior antibiotic treatment for a UTI within 6 months before study inclusion (odds ratio, 2.45; 95% CI, 1.25-4.88; *P* = .01); no associations were found for age, sex, body mass index, CIC frequency, neurogenic cause, or comorbidities (diabetes, chronic obstructive pulmonary disease, cardiovascular disease, or hypertension).

### Adherence to Protocol

In the reusable catheter group, single-use catheters were permitted for up to 20% of weekly catheterizations. Adherence to the assigned intervention was high, with reusable catheters used for a mean (SD) of 88.6% (19.0%) of catheterizations in the ITT population, 91.9% (9.4%) in the mITT population, and 92.6% (8.7%) in the per-protocol population. Of the 113 patients (58.5%) in the reusable catheter group who completed 1 year of follow-up, 105 (92.9%) preferred to continue using the reusable catheter after the study.

### Primary Outcome

In the mITT population (n = 326), the mean (SD) follow-up was 12.0 (1.9) months in the single-use group and 11.4 (2.9) months in the reusable group. Of the total 211 UTIs during the study, 147 (69.7%) were culture confirmed and 52 (24.6%) were diagnosed based on sediment or dipslide. One or more UTIs occurred in 59 of 191 patients (30.9%) in the single-use group and 40 of 134 (29.9%) in the reusable group. The incidence rate was 0.054 UTIs per patient-month (95% CI, 0.040-0.069) in the single-use group and 0.050 (95% CI, 0.035-0.067) in the reusable group, with an absolute difference of −0.004 (95% CI, −0.025 to 0.019), meeting the prespecified noninferiority margin. These results were consistent across all sensitivity analyses (Table 2), including cluster-robust analyses accounting for potential within-site correlation.

**Table 2.** Incidence Rate of Urinary Tract Infections Per Patient-Month in the Modified Intention-to-Treat, Intention-to-Treat, and Per-Protocol Populations

Study population	No. of participants included in analysis	Follow-up, mean (SD), mo	Incidence rate (95% CI)	Absolute difference (95% CI)^a^	Incidence rate ratio (95% CI)
**Modified intention to treat^b^**					
Single use	191	12.0 (1.9)	0.054 (0.040 to 0.069)	−0.004 (−0.025 to 0.019)	0.87 (0.55 to 1.37)
Reusable	134	11.4 (2.9)	0.050 (0.035 to 0.067)		
**Intention to treat^c^**					
Single use	192	12.0 (2.0)	0.054 (0.039 to 0.069)	−0.002 (−0.028 to 0.026)	0.87 (0.56 to 1.36)
Reusable	185	8.6 (5.2)	0.052 (0.032 to 0.079)		
**Per protocol^d^**					
Single use	177	12.5 (0.6)	0.05 (0.036 to 0.065)	−0.01 (−0.032 to 0.011)	0.79 (0.48 to 1.31)
Reusable	113	12.5 (0.4)	0.04 (0.025 to 0.057)		

^a^ Unadjusted absolute difference in incidence rate.

^b^ Modified intention-to-treat population (primary analysis) includes all patients with at least 6 weeks of follow-up data (first clinical visit).

^c^ Intention-to-treat population includes all randomized patient who initiated treatment regardless of protocol adherence.

^d^ Per-protocol population includes patients who completed the 12-month follow-up according to the study protocol.

**Figure 2.**
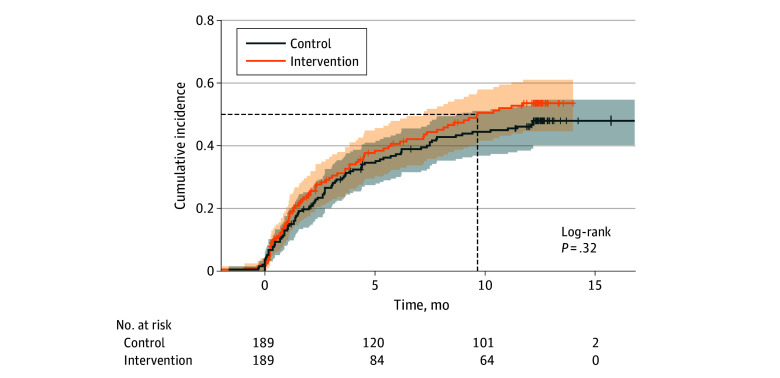
Kaplan-Meier Plot of Time to First Urinary Tract Infection by Treatment Group (Single-Use vs Reusable Catheters) Cumulative incidence of first urinary tract infection is shown for patients randomized to reusable (intervention) or single-use (control) catheters. Shaded areas represent 95% CIs. Patients who discontinued or were lost to follow-up were censored at the time of last contact. Differences between groups were evaluated using the log-rank test.

Missing UTI data were minimal, observed in 12 participants in the ITT population and 5 participants in the mITT population (both <5%). The worst-case sensitivity analysis, substituting missing values in the reusable catheter arm with the maximum observed UTI rate, did not alter the noninferiority conclusion (absolute difference, −0.003; 95% CI, −0.024 to 0.020). To account for the higher dropout rate in the reusable group, a CACE analysis was performed, yielding an unadjusted estimate of 0.016 UTIs per patient-month (95% CI, −0.126 to 0.158; *P* = .83), with a strong instrument (*F* = 35.3; *P* < .001); the nonsignificant Wu-Hausman test result indicated that nonadherence did not meaningfully bias the primary mITT result, confirming robustness of the noninferiority conclusion (eTable 2 in Supplement 2). The cumulative incidence of first UTI did not differ significantly between groups over time (control group: 90/189 events; intervention group: 86/189 events; log-rank *P* = .32) (Figure 2).

### Secondary Outcomes

Catheter-related complications were analyzed in the ITT population, which included all participants who initiated the assigned intervention (Table 3). The proportion of patients experiencing 1 or more UTI was 31.0% (59 of 192) in the single-use group and 27.0% (43 of 185) in the reusable group (*P* = .52). UTI-related hospitalizations occurred in 3 patients (1.6%) in each group. Bacteremic UTIs were rare, with 1 case (0.5%) reported in the reusable group. Systemic infections, including pyelonephritis, epididymo-orchitis, and prostatitis, were infrequent and did not differ significantly between groups. Macroscopic hematuria was observed in 12.0% of patients in both groups, with a median (IQR) of 2 (1-4) episodes in the single-use group and 1 (1-2) episode in the reusable group. Urethral strictures were rare and equally distributed (0.5% in both groups). No cases of urolithiasis were reported in either group.

**Table 3.** Catheter-Related Complications and AEs by Treatment Group (Single-Use vs Reusable Catheters)

Complication or AD	No. (%) of participants^a^		*P* value
	Single-use catheter (n = 192)^b^	Reusable catheter (n = 185)^b^	
Catheter-related complications			
≥1 UTI	59 (31.0)	43 (27.0)	.53
No. of UTIs, median (IQR)^c^	2 (1-2.5)	1 (1-2)	NA
Bacteremic UTI	0	1 (0.5)	>.99
Hospitalization due to UTI	3 (1.6)	3 (1.6)	>.99
Pyelonephritis	1 (0.5)	1 (0.5)	>.99
Epididymo-orchitis	4 (2.1)	2 (1.1)	.65
Prostatitis	1 (0.5)	0	>.99
Macroscopic hematuria	24 (12.0)	23 (12.0)	.53
No. of macroscopic hematuria episodes, median (IQR)^c^	2 (1-4)	1 (1-2)	NA
Urethral stricture	1 (0.5)	1 (0.5)	>.99
Urolithiasis	0	0	>.99
AEs			
CTCAE grade^d^			
1	11 (5.5)	39 (21.1)	<.001
2	3 (1.6)	5 (2.7)	
3	17 (8.9)	5 (2.7)	
4	1 (0.5)	2 (1.1)	
5	0	0	
AEs grades 1-2^d^			
No. of AEs per participant, median (IQR)^c^	1 (1-1)	1 (1-1)	NA
Urethral irritation	9 (4.7)	37 (20.0)	.04
Abdominal pain	NA	1 (0.5)	NA
Vaginal atrophy	NA	2 (1.1)	NA
Bladder spasms	NA	1 (0.5)	NA
Skin lesion due to cleaning solution	NA	1 (0.5)	NA
Sacral pressure ulcer	NA	1 (0.5)	NA
Choledocholithiasis	NA	1 (0.5)	NA
Urinary incontinence	1 (0.5)	NA	NA
Limb weakness	1 (0.5)	NA	NA
TIA	1 (0.5)	NA	NA
Knee fracture	1 (0.5)	NA	NA
Hand laceration	1 (0.5)	NA	NA
SAEs grade ≥3^d^	14 (7.0)	7 (4.0)	NA
No. of SAEs per participant, median (IQR)^c^	1 (1-1)	1(1-1)	NA
Study-related SAEs	3 (1.6)	3 (1.6)	NA
Non–study-related SAEs	15 (7.8)	4 (2.2)	NA

Abbreviations: AE, adverse event; CTCAE, Common Terminology Criteria for Adverse Events; NA, not applicable; SAE, serious adverse event; TIA, transient ischemic attack; UTI, urinary tract infection.

^a^ Unless otherwise indicated.

^b^ Catheter-related complications and AEs were analyzed in the intention-to-treat population, defined as all participants who initiated the assigned intervention.

^c^ Median (IQR) values were calculated only among participants with 1 event or more.

^d^ AEs were graded according to the CTCAE, version 5.0, where grade 1 indicates mild; grade 2, moderate; grade 3, severe; grade 4, life-threatening; and grade 5, death related to AE. SAEs were defined as events of grade 3 or higher.

At week 52, catheter-related quality of life (Intermittent Self-Catheterization Questionnaire) was significantly higher in the reusable catheter group (mean difference, 3.6; 95% CI, 0.3-7.0; *P* = .03), whereas catheterization satisfaction (Intermittent Catheterization Satisfaction Questionnaire) was significantly higher in the single-use group (mean difference, −0.12; 95% CI, −0.22 to −0.01; *P* = .03). Health-related quality of life, assessed with the EuroQoL 5-dimension 5-level (EQ-5D-5L) questionnaire and the Qualiveen Short Form, was similar between groups. The mean EQ-5D-5L health utility score was 0.78 in both arms (mean difference, 0.00; 95% CI, −0.04 to 0.05; *P* = .91), and the mean Qualiveen Short Form score was 1.41 (95% CI, 1.33-1.49) in the single-use group and 1.40 (95% CI, 1.31-1.49) in the reusable group (mean difference, −0.02; 95% CI, −0.14 to 0.11; *P* = .81).

### Adverse Events

AEs were analyzed in the ITT population, which included all participants who initiated the assigned intervention (Table 3). A total of 26 patients (13.5%) in the single-use group and 50 patients (27.0%) in the reusable group experienced 1 or more AE. The overall distribution of CTCAE grades differed significantly between groups (control vs intervention groups: grade 1, 11 [5.5%] vs 39 [21.1%]; grade 2, 3 [1.6%] vs 5 [2.7%]; grade 3, 17 [8.9%] vs 5 [2.7%]; grade 4; 1 [0.5%] vs 2 [1.1%]; grade 5, 0 vs 0; Fisher exact test *P* < .001). Among those with 1 or more AE, the median (IQR) number of events was 1 (1-1) in both groups. Low-grade AEs (grades 1–2) were more frequent in the reusable catheter group, mainly due to urethral irritation (37 [20.0%] vs 9 [4.7%], *P* = .04). Initially, 60 of 185 participants (32.4%) used additional lubricant, but this decreased to 10 of 113 (8.8%) after 1 year in the per-protocol population. Lubricant use at baseline did not differ significantly between those who dropped out and those who continued the study (25 of 70 [35.7%] [10 unknown] and 35 of 113 [31.0%]; *P* = .51). Other low-grade events reported in the reusable group included abdominal pain (of unclear cause), vaginal atrophy, bladder spasms, and a skin lesion caused by the cleaning solution.

The rates of SAEs (grade 3 or higher) were low and comparable between the groups. Study-related SAEs were similar in both groups and included 3 cases (1.6%) of hospitalization due to UTIs. Non–study-related SAEs occurred in 15 patients (7.8%) in the single-use group and 4 patients (2.2%) in the reusable group. These included diagnosis such as ileus, cholecystitis, pneumonia, and femur fracture. No grade 5 events were reported.

## Discussion

In this multicenter randomized clinical trial, reusable catheters were noninferior to single-use catheters for CIC regarding UTI incidence, with comparable rates of serious complications. However, the high dropout rate in the reusable group (39.0%), driven by ease-of-use challenges and urethral irritation, and mixed patient-reported outcomes suggest that reusable catheters are not suitable for all patients.

The COMPARE trial builds on a limited body of evidence because previous randomized clinical trials comparing reusable and single-use catheters were small and underpowered. Previously, 8 small randomized clinical trials have compared UTI rates between single-use and reusable catheters, with the largest including 80 participants.^14^ Trial durations ranged from 8 weeks to 12 months, with no significant differences in UTI rates observed.^14^ A meta-analysis^15^ pooling these data found no significant difference in UTI incidence between catheter types (relative risk, 0.91; 95% CI, 0.66-1.27; *P* = .59). Similarly, a recent Cochrane review^6^ concluded that the evidence was inconclusive, citing low-certainty data with CIs consistent with both benefit and harm (relative risk, 0.98; 95% CI, 0.55-1.74; 2 studies; 97 participants). AEs were rarely reported; one trial reporting no events in either group, and no additional data on safety or patient preferences were provided.^16^

In our trial, catheter-related complications and AEs were generally comparable between groups, and SAEs, including UTI-related hospitalizations and systemic infections, were rare. However, urethral irritation during catheter insertion or removal was significantly more frequent in the reusable catheter group (20.0% vs 4.7%), representing a clinically important finding that should not be minimized because it can affect comfort, adherence, and quality of life in patients relying on long-term catheterization. This likely reflects the uncoated surface of reusable catheters; hydrophilic-coated single-use catheters reduce friction and have been associated with less urethral microtrauma and stricture formation.^17^ Similar use of additional lubricant who continued vs discontinued reusable catheters suggests that lubrication alone does not sufficiently mitigate irritation. Interestingly, a Cochrane review by Pietro et al^6^ suggested that uncoated catheters may be associated with a slightly lower risk of urethral trauma and bleeding compared with hydrophilic-coated catheters (relative risk, 1.37; 95% CI, 1.01-1.87). Consistent with previous randomized clinical trials^18,19^ comparing uncoated and hydrophilic-coated catheters, this study found no difference in macroscopic hematuria between groups. Urethral irritation in the reusable catheter group may also have been influenced by factors beyond catheter coating. Participants frequently reported difficulties with insertion due to insufficient catheter stiffness, which may reduce control and increase friction during use. Additionally, sharper edges (eyelets) or residual cleaning agent despite rinsing before use could have contributed to mucosal irritation. These findings highlight the need for continued refinement of reusable catheter design to reduce urethral irritation and improve usability.

The discontinuation rate was high in the reusable group (39.0%), particularly during the first 6 weeks. Patients with a history of frequent UTIs in the 6 months before enrollment were more likely to discontinue, possibly reflecting concerns about infection risk. Additional barriers included difficulties with ease of use and the perceived burden of repeated rinsing and disinfectant replacement. These findings suggest that reducing the complexity of the cleaning protocol may be key to improving adherence. Evidence from the study by Takahashi et al^20^ and long-term experience from Thailand^21^ indicate that simplified cleaning protocols, without daily rinsing or frequent solution replacement, can maintain safety without increasing UTI risk. Optimizing both catheter design and cleaning protocols will be crucial to improving usability and supporting broader clinical adoption in the future.

Anthropogenic climate change is one of the greatest global health threats of the 21st century,^22^ with the health sector accounting for nearly 5% of global greenhouse gas emissions.^23^ During the past 2 decades, the use of single-use catheters has increased by approximately 300%,^24^ contributing to substantial environmental and financial burdens. In the Netherlands, expenditures on intermittent catheters increased from €16.4 million in 1997 to €74.6 million in 2018 (to convert euros to US dollars, multiply by 1.17).^24^ Transitioning to reusable catheters could meaningfully reduce environmental burden, potentially preventing millions of kilograms of plastic waste globally each year. On the basis of mixed use, patients would need approximately 26 to 200 catheters per year compared with 1825 single-use catheters. This approach would also improve affordability and access in low-income countries, where single-use catheters remain cost-prohibitive.

Additional advantages of reusable catheters include greater patient autonomy and reduced concerns about catheter shortages. Several health care insurances reimburse up to 4 catheters per day, which may not meet all patients’ needs, potentially causing stress and negatively impacting quality of life. Furthermore, the need to store and transport large quantities of single-use catheters during travel poses practical challenges. Although some participants discontinued reusable catheters due to ease-of-use challenges, 58.5% continued using reusable catheters and 92.9% of those preferred to keep using them post-study. Catheter-related quality of life favored the reusable group, whereas catheterization satisfaction favored single-use catheters, suggesting that patient experience is multidimensional and that neither catheter type is universally superior. These findings support a complementary rather than replacement role for reusable catheters, consistent with qualitative evidence that most users prefer combine catheter types depending on the situation.^25,26^ A flexible, patient-centered approach, offering reusable catheters as an additional option rather than a substitute, may therefore best serve individual needs. Product innovation remains essential to improve usability and broaden acceptability.

### Strengths and Limitations

This randomized noninferiority trial has several strengths, including a large and diverse CIC population encompassing both neurogenic and nonneurogenic causes recruited across multiple centers to enhance generalizability. Validated outcome measures, including a standardized UTI definition, were applied, and a rigorous cleaning protocol was implemented for reusable catheters. Despite a 39.0% dropout rate in the reusable catheter group, most participants who continued with the study demonstrated high adherence, indicating the feasibility of reusable catheters in community settings.

This study also has limitations. An important limitation is the high dropout rate in the reusable catheter group, which may introduce attrition bias and limit generalizability, while also highlighting practical challenges and user burden that can guide future design improvements. A CACE analysis supported the primary findings, mitigating concerns about attrition bias. Additionally, although UTI diagnoses were based on standardized criteria and supported by urine testing, microbiological confirmation by culture was not consistently obtained. Although this would have strengthened the study from a microbiological perspective, the current data reflect clinical practice, particularly in primary care settings.

## Conclusions

In this randomized clinical trial of patients with urinary retention, reusable catheters were noninferior to single-use catheters for CIC with respect to UTIs. However, the substantially higher discontinuation rate (39.0%), increased urethral irritation (20.0% vs 4.7%), and mixed PROs temper the strength of this conclusion and indicate that reusable catheters are not suitable for all patients. Nevertheless, they can be offered as an additional option based on individual patient preference and circumstances. Given their potential to reduce health care costs, reduce plastic medical waste, and improve access in low-resource settings, continued investment in design improvements is essential to enhance tolerability and facilitate broader implementation.

## Notes

Supplement 1.
Trial Protocol and Statistical Analysis Plan

Supplement 2. **eTable 1. **Summary of Analysis Populations **eTable 2. **Unadjusted Complier Average Causal Effect (CACE) Estimates for the Modified Intention-to-Treat and Intention-to-Treat Populations

Supplement 3.
Data Sharing Statement
