# Supplementary material for: Single-Use vs Reusable Catheters for Intermittent Catheterization in Patients With Urinary Retention: The COMPARE Randomized Clinical Trial
Source: JAMA Netw Open. 2026 Jun 30;9(6):e2620871. doi: 10.1001/jamanetworkopen.2026.20871 (PMC13320647; doi:10.1001/jamanetworkopen.2026.20871)
Supplement: Supplement 1. — Trial Protocol and Statistical Analysis Plan [file jamanetwopen-e2620871-s001.pdf]

Single use vs reusable catheters in intermittent CatheterizatiOn for treatment of urinary retention: a Multicenter, Prospective, RandomizEd controlled, non-inferiority trial (COMPaRE)

Version 5.0

3 October 2023

PROTOCOL TITLE 'Single use vs reusable catheters in intermittent CatheterizatiOn for treatment of urinary retention: a Multicenter, Prospective, RandomizEd controlled, non-inferiority trial (COMPaRE)'

**Protocol ID**	NL68597.078.19 / OZBS62.18167
**Short title**	COMPaRE
**EudraCT number**	*Not applicable*
**Version**	5.0
**Date**	3 October 2023
**Coordinating investigator/project leader/Principal investigator**	Dr. B.F.M. Blok, Urologist<br>Dept. Urology<br>Erasmus MC, room Na-1510<br>Dr. Molewaterplein 40<br>3015 GD Rotterdam<br>Tel: +31 (0) 10 – 703 10 39<br>b.blok@erasmusmc.nl
**Sub-investigators**	Dr. J.R. Scheepe, Pediatric urologist<br>Dept. Urology<br>Erasmus MC, room Na-1508<br>Dr. Molewaterplein 40<br>3015 GD Rotterdam<br>Tel: +31 (0) 10 – 703 65 59<br>j.scheepe@erasmusmc.nl<br><br>Drs. F.E.E. van Veen, PhD candidate<br>Dept. Urology<br>Erasmus MC, room Na-1524<br>Dr. Molewaterplein 40<br>3015 GD Rotterdam<br>Tel: +31 (0) 10 – 703 35 71<br>f.vanveen@erasmusmc.nl
**Sponsor**	Erasmus MC, Dept. Urology
**Subsidising party**	ZonMW

**Independent expert**	Drs. J. van den Hoek<br>Pediatric urologist<br>Dept. Urology<br>Erasmus MC, room Na-1508<br>Dr. Molewaterplein 40<br>3015 GD Rotterdam<br>Tel: +31 (0) 10 – 703 93 34
**Laboratory sites**	Not applicable
**Pharmacy**	Not applicable

PROTOCOL SIGNATURE SHEET VERSION 5.0

Name	Signature	Date
**Head of Department of Urology:**<br>*Dr. J.L. Boormans*		
**Coordinating Investigator/Project leader/Principal Investigator:**<br>*Dr. B.F.M. Blok*		

TABLE OF CONTENTS

1. INTRODUCTION AND RATIONALE 10
2. OBJECTIVES 12
3. STUDY DESIGN 13
4. STUDY POPULATION 15
4.1 Population (base) 15
4.2 Inclusion criteria 15
4.3 Exclusion criteria 15
4.4 Sample size calculation 15
5. TREATMENT OF SUBJECTS 18
5.1 Investigational product 18
5.2 Use of co-intervention 18
5.3 Escape medication 18
6. INVESTIGATIONAL PRODUCT 19
6.1 Name and description of investigational product 19
6.2 Summary of findings from non-clinical studies 19
6.3 Summary of findings from clinical studies 19
6.4 Summary of known and potential risks and benefits 20
6.5 Description and justification of route of administration and dosage 20
6.6 Dosages, dosage modifications and method of administration 21
6.7 Preparation and labelling of Investigational Medicinal Product 21
6.8 Drug accountability 21
7. NON-INVESTIGATIONAL PRODUCT 22
7.1 Name and description of non-investigational product(s) 22
7.2 Summary of findings from non-clinical studies 22
7.3 Summary of findings from clinical studies 22
7.4 Summary of known and potential risks and benefits 22
7.5 Description and justification of route of administration and dosage 22
7.6 Dosages, dosage modifications and method of administrationNot applicable 22
7.7 Preparation and labelling of Non Investigational Medicinal Product 22
7.8 Drug accountability 22
8. METHODS 23
8.1 Study parameters/endpoints 23
8.1.1 Main study parameter/endpoint 23
8.1.2 Secondary study parameters/endpoints 23
8.1.3 Other study parameters 24
8.2 Randomisation, blinding and treatment allocation 24
8.3 Study procedures 24
8.4 Withdrawal of individual subjects 25
8.4.1 Specific criteria for withdrawal 25
8.5 Replacement of individual subjects after withdrawal 25
8.6 Follow-up of subjects withdrawn from treatment 25

8.7 Premature termination of the study 25
9. SAFETY REPORTING 26
9.1 Temporary halt for reasons of subject safety 26
9.2 AEs, SAEs and SUSARs 26
9.2.1 Adverse events (AEs) 26
9.2.2 Serious adverse events (SAEs) 26
9.2.3 Suspected unexpected serious adverse reactions (SUSARs) 27
9.3 Annual safety report 27
9.4 Follow-up of adverse events 28
9.5 Data Safety Monitoring Board (DSMB) / Safety Committee 28
10. STATISTICAL ANALYSIS 29
10.1 Primary study parameters 29
10.2 Secondary study parameters 29
10.3 Other study parameters 30
10.4 Interim analysis 30
11. ETHICAL CONSIDERATIONS 31
11.1 Regulation statement 31
11.2 Recruitment and consent 31
11.3 Objection by minors or incapacitated subjects 31
11.4 Benefits and risks assessment, group relatedness 31
11.5 Compensation for injury 31
11.6 Incentives 32
12. ADMINISTRATIVE ASPECTS, MONITORING AND PUBLICATION 33
12.1 Handling and storage of data and documents 33
12.2 Monitoring and Quality Assurance 33
12.3 Amendments 33
12.4 Annual progress report 33
12.5 Temporary halt and (prematurely) end of study report 33
12.6 Public disclosure and publication policy 34
13. STRUCTURED RISK ANALYSIS 35
13.1 Potential issues of concern 35
13.2 Synthesis 35
14. REFERENCES 36

LIST OF ABBREVIATIONS AND RELEVANT DEFINITIONS

**ABR**	**General Assessment and Registration form (ABR form), the application form that is required for submission to the accredited Ethics Committee; in Dutch: Algemeen Beoordelings- en Registratieformulier (ABR-formulier)**
**AE**	**Adverse Event**
**AR**	**Adverse Reaction**
**CA**	**Competent Authority**
**CCMO**	**Central Committee on Research Involving Human Subjects; in Dutch: Centrale Commissie Mensgebonden Onderzoek**
**CV**	**Curriculum Vitae**
**DSMB**	**Data Safety Monitoring Board**
**EU**	**European Union**
**EudraCT**	**European drug regulatory affairs Clinical Trials**
**GCP**	**Good Clinical Practice**
**GDPR**	**General Data Protection Regulation; in Dutch: Algemene Verordening Gegevensbescherming (AVG)**
**IB**	**Investigator's Brochure**
**IC**	**Informed Consent**
**IMP**	**Investigational Medicinal Product**
**IMPD**	**Investigational Medicinal Product Dossier**
**METC**	**Medical research ethics committee (MREC); in Dutch: medisch-ethische toetsingscommissie (METC)**
**(S)AE**	**(Serious) Adverse Event**
**SPC**	**Summary of Product Characteristics; in Dutch: officiële productinformatie IB1-tekst**
**Sponsor**	**The sponsor is the party that commissions the organisation or performance of the research, for example a pharmaceutical company, academic hospital, scientific organisation or investigator. A party that provides funding for a study but does not commission it is not regarded as the sponsor, but referred to as a subsidising party.**
**SUSAR**	**Suspected Unexpected Serious Adverse Reaction**
**UAVG**	**Dutch Act on Implementation of the General Data Protection Regulation; in Dutch: Uitvoeringswet AVG**
**WMO**	**Medical Research Involving Human Subjects Act; in Dutch: Wet Medisch-wetenschappelijk Onderzoek met Mensen**

SUMMARY

Rationale: Clean intermittent catheterization (CIC) is the treatment of choice for patients suffering from idiopathic or neurogenic urinary retention. Possible causes are enlarged prostate, pelvic surgery, spinal cord injury (SCI) or multiple sclerosis (MS). Most patients catheterize four to six times a day, keeping the catheterized volume preferably below 400-500 ml. Virtually all patients on CIC in the Netherlands utilize single use (=disposable) catheters, which is in contrast to the practice of the use of reusable catheters in many non-European countries. The main reason is that there exists thus far no CE marked reusable catheter. Our group has found that a Japanese company owns the CE mark and this will be used to introduce the reusable catheter in Europe. The suppliers who make the single use catheters support all patient advocate and continence nurse societies. The webpages of the industry suggests strongly that disposable catheters are less associated with urinary tract infections and other complications. In this context, it is relevant to note that the global urinary catheter market size was valued in 2015 at USD 3.4 billion, with gradual grow in future perspective. This market is formed for around 60% by disposable catheters. The available literature on the differences in safety and efficacy between single use and reusable catheters is conflicting. On the one hand, it has been suggested with in vitro experiments that reuse of catheters introduces unwanted bacterial contamination and therefore increases the risk of symptomatic urinary infections and other complications, like stone formation and urethral strictures. On the other hand, limited evidence in patients on CIC suggest that reusable catheters are not less safe and not less effective as disposable catheters. The research question of this study will be: ‘Is the use of reusable catheters in patients on intermittent catheterization in urinary retention not less safe or not less efficient as the use of single use catheters?’. The potential effects on the Dutch health economics will be provided. The project is supported by ZonMW (project 853001104 in the framework of ‘Goed Gebruik Hulpmiddelenzorg’), granted in September 2018.

Objective: The main objective of this trial is to determine whether reusable catheters are not less safe as single use catheters, measured by symptomatic UTIs. Secondary objectives are adverse events like hospital admissions due to UTIs, changes in urine cultures/urinary microbiome, urethral damage/strictures, kidney/bladder stone formation and quality of life of the participants. Cost effectiveness and recommendations for practice will be provided.

Study design: The study will be performed in patients who CIC in a multicenter, prospective, randomized controlled, non-inferiority trial. The patients on CIC will be assigned to either

single use or reuse catheterization during twelve months with the primary outcome ‘the amount of symptomatic UTIs’. The total duration of this trial is 36 months.

Study population: Patients who self-catheterize and meet up to the inclusion criteria will be screened, included and randomized at the outpatient clinic of the participating hospitals. A total of 386 patients is needed to be included to meet up to the sample size calculation.

Intervention: Patients will be randomized into two groups, one group will use the single use catheters, the other group will start using the reusable catheter which will be renewed every two weeks.

Main study parameters/endpoints: The primary outcome is symptomatic urinary tract infections (UTIs). Secondary outcomes will be other adverse events, like hospital stays due to UTIs, bladder stones, haematuria, changes in urine cultures/urinary microbiome and symptom specific and quality of life questionnaires. Quality of life will be measured by the following validated questionnaires: EQ-5D-5L and SF-Qualiveen. The ease of use of the catheters will be evaluated by the Patient Global Impression of Improvement (PGI-I) scale, the ISC-Q and the InCaSaQ. Cost-effectiveness calculation on both types of catheters will be made. The economic evaluation will be done using the iMCQ and iPCQ questionnaires. Two additional questions concerning patients thoughts on environmental burden and healthcare costs will be asked at week 0 and week 52.The questionnaires will be send to the patients by email using Gemstracker/Limesurvey.

Nature and extent of the burden and risks associated with participation, benefit and group relatedness: Patients will have an extension of two clinical visits at the hospital and seven telephone contacts for follow up of the trial. The extra burden for patients in the reusable catheter group is the cleaning procedure of the catheter in which patients will be schooled. Prior to the four clinical visits, patients will fill in the above mentioned questionnaires which will be send by email using Gemstracker/Limesurvey.

1. INTRODUCTION AND RATIONALE

Millions of people have difficulty in emptying their urinary bladder resulting in urinary retention or clinically significant post void residue (PVR) (1). This retention or residue is due to lower urinary tract dysfunction, in which the cause is usually unknown (idiopathic) or caused by well-known diseases like spinal cord injury (SCI) or multiple sclerosis (MS). To empty the bladder, the treatment of choice is clean intermittent self-catheterization (CIC) or, clinically less preferred, an indwelling catheter. Patients administer CIC 4-6 times a day, keeping the catheterized volume preferably below 400-500 ml (2, 3). Virtually all patients on CIC in the Netherlands utilize single use (=disposable) catheters, which is in contrast to the practice of the use of reusable catheters in many non-European countries. The main reason is that there exists thus far no CE marked reusable catheter on the European market. The suppliers who make the single use catheters widely support patient advocate groups, continence nurse societies and relevant physicians societies. The webpages of the industry suggests strongly that disposable catheters are less associated with urinary tract infections and other complications. In this context, it is relevant to note that the global urinary catheter market size was valued in 2015 at USD 3.4 billion, with gradual growth in future perspective. The majority of this market is formed by intermittent disposable catheters, which are accountable for around 60% of the market (4). The current guideline of the European Association of Urology Nurses (EAUN) on intermittent catheterization discusses the possible advantage in favor of the single use catheters based on low (grade 4) level of evidence, mainly concerning the efficacy of cleaning catheters by different methods (5). Other guidelines from the European Urology Association (EAU) and Verenso do not discuss differences between single use and reusable catheters for CIC (3, 6). The available literature on the differences in safety and efficacy between single use and reusable catheters is conflicting and of low level of evidence. On the one hand, it has been suggested that reuse of catheters introduces unwanted bacterial contamination and therefore increases the risk of symptomatic urinary infections and other complications, like stone formation and urethral strictures (7). On the other hand, evidence in patients on CIC suggest that reusable catheters are as safe and effective as disposable catheters (8). Prieto et al described in their Cochrane analysis of 2014 that there exists no evidence for differences on the incidence of UTI in patients using reusable catheters compared to patients using single-use catheters (9). This review was forced to withdrawn in 2017 with the argument that more fundamental research was necessary to obtain high level evidence (10). Consulting physicians are willing to prescribe reusable catheters or a mixture of single-use and reusable, if the use is substantiated by evidence (11). In view of the lack of this evidence, clinical research is recommended to investigate if the use of multi-use (reusable) catheters is not less safe than

disposable (single-use) catheters. (8, 12). In the Netherlands the use of single-use catheters for CIC is almost 100%. In contrast, the use of reusable catheters in countries outside Europe is much higher, possibly due to restrains in healthcare budget (12).
Possible advantages of reusable catheters include health care cost efficiency. Potential savings are calculated globally (see Appendix ‘Potential Savings) if 50% of all patients in the Netherlands start using the reusable catheter. The costs of the single use catheters vary widely. For this calculation two prices were used (1.03 and 1.70 euro’s per catheter). The potential savings vary from 13.184.437,50 to 23.272.125,00 euro’s a year in the Netherlands. Other possible advantages of reusable catheters include environmental advantages. The majority of patients feels a growing responsibility for environmental consequences of the use of catheters and keeping healthcare affordable, as do patients that are on CIC.
The potential environmental waste savings are also calculated globally (see Appendix ‘Potential environmental waste savings’) if 50% of all patients in the Netherlands start using a reusable catheter. The potential average environmental waste savings is estimated on 371.480 kg/year in the Netherlands, which is a possible reduction of 48,7% of all waste generated by single use catheters in the Netherlands. This calculation is based on the study of Sun et al, which investigated the environmental aspects of single use catheters in the United states (13).
The last possible advantages of the reusable catheters, but equally important, include more patient choice and reducing fear of running out of catheters. Several healthcare insurances, provide up to four catheters a day, which is often not sufficient for the needs of all patients. This introduces potential stress for the patients and does not stimulate the health of the individual patient. Also, it is clear that storage of large amounts of catheters, or travelling for vacation with a stock of needed catheters, is not ideal for patients and gives fear of running out of catheters.

Our group has found that a CE-certified Japanese manufacturer designed a reusable catheter. This catheter will be used to introduce the reusable catheter in Europe through this study.

2. OBJECTIVES

The aim of this trial is to compare single use vs reusable catheters in clean intermittent catheterization and to find out if reusing catheters is not less safe and not less efficient as the current single use practice, leading to the following objectives:

Primary Objective:

1. To determine whether reusable catheters are at least not less safe as single use catheters, measured by symptomatic UTIs.

Secondary Objectives:

1. To register hospital admissions due to UTIs or other adverse events due to CIC.
2. To register other adverse events like the number of urethral damage/strictures and kidney/bladder stone formation in both groups.
3. To determine whether reuse of catheters leads to changes of the urine cultures/urinary microbiome (in cooperation with the department of Medical Microbiology).
4. To perform economical evaluation of the cost-effectiveness of single use versus reusable catheters.
5. To explore patients' perspective on ease of use and cleaning of the reusable catheters compared to the single use catheters.
6. To formulate conclusive recommendations for health care providers and re-formulations of existing protocols.

3. STUDY DESIGN

This study will be a prospective non-inferiority trial with two randomized groups. The patients will be screened, included and randomized at the outpatient clinic of the participating hospitals. One group of patients will use the reusable catheter and the control group will use the single use catheters. After inclusion, the patients will be followed up for a period of twelve months.

The following schedule will be used:

Week	Telephone/clinical visit	What	Questionnaires
Week 0, *baseline*	Clinical visit 1	- Inclusion of patients<br>- Informed consent signing<br>- Randomization into two groups:<br>*Intervention group (reusable) N = 228*<br>Or<br>*Control group (single use) N = 228*<br>- If randomized in intervention group, schooling on how to use the reusable catheter is provided by the continence nurse<br><br>- Urinary sample will be obtained for testing	- EQ-5D-5L<br>- SF-Qualiveen<br>- ISC-Q<br>- IncasaQ<br>- Two opinion questions about the environmental and financial burden of catheters
Week 1	Telephone call	Evaluation of catheter use, patients will be asked for adverse events and possible risen questions will be answered.	
Week 6	Telephone call	To evaluate the first impression, evaluate if the cleaning routine of the catheter is correctly preformed, asked for adverse events through standardized questions in Gemstracker/LimeSurvey and possible risen questions will be answered.	- EQ-5D-5L<br>- SF-Qualiveen<br>- ISC-Q<br>- IncasaQ<br>- iMCQ<br>- iPCQ<br>- PGI-I
Week 12	Telephone call	The use of both types of catheters will be evaluated, patients will be asked for adverse events through standardized questions in Gemstracker/LimeSurvey and possible risen questions will be answered.	
Week 18	Telephone call	The use of both types of catheters will be evaluated, patients will be asked for adverse events through standardized questions in Gemstracker/LimeSurvey and possible risen questions will be answered.	

Week 26	Telephone call	To evaluate the first six months of usage of the reusable catheter and the persistent use of the single use catheter, evaluate if the cleaning routine of the catheter is correctly preformed. Patients asked for adverse events through standardized questions in Gemstracker/LimeSurvey and possible risen questions will be answered.	- EQ-5D-5L<br>- SF-Qualiveen<br>- ISC-Q<br>- IncasaQ<br>- iMCQ<br>- iPCQ<br>- PGI-I
Week 34	Telephone call	The use of both types of catheters will be evaluated, patients will be asked for adverse events through standardized questions in Gemstracker/LimeSurvey and possible risen questions will be answered.	
Week 42	Telephone call	The use of both types of catheters will be evaluated, patients will be asked for adverse events via standardized questions in Gemstracker/LimeSurvey and possible risen questions will be answered.	
Week 52, *endpoint*	Clinical visit 2	After completing the twelve months of follow up, patients will be asked one last time for adverse events via standardized questions in Gemstracker/Limesurvey. Additionally the trial will be evaluated.<br><br>- Urinary samples will be obtained for testing	- EQ-5D-5L<br>- SF-Qualiveen<br>- ISC-Q<br>- IncasaQ<br><br>- iMCQ<br>- iPCQ<br>- PGI-I<br>- Two opinion questions about the environmental and financial burden of catheters

All questionnaires will be sent to the patients by email prior to the contact moment by Limesurvey/Gemstracker.

4. STUDY POPULATION

4.1 Population (base)

Patients will be included at the outpatients clinic of the urology department of the participating centres. There are several possible causes why patients have to self-catheterize. As described above, urinary retention or post void residue can be caused by urinary tract dysfunction due to idiopathic or neurogenic urinary retention. All included patients are able to self-catheterize and are experienced for at least two weeks.

4.2 Inclusion criteria

In order to be eligible to participate in this study, a subject must meet all of the following criteria:

- Male/Female patients ≥ 16 years old
- Diagnosed with urinary retention or significant post-void residue due to non-neurogenic or neurogenic causes
- Expected chronic, but at least for a duration of twelve months, necessity for daily drainage of the urinary bladder
- Be able to administer self CIC via the urethra daily and have at least two weeks of experience in CIC.

4.3 Exclusion criteria

A potential subject who meets any of the following criteria will be excluded from participation in this study:

- Age < 16 years
- Temporary use of catheterization because of transient causes
- Known significant urethral stricture which prevents CIC
- Urinary tract stones
- Bladder augmentation
- Non-urethral catheterization
- History of bladder cancer with active follow-up
- The use of immunosuppressives for transplantation or auto-immune diseases
- Neurocognitive disease which prevents complete comprehension of the study

4.4 Sample size calculation

The number of studies that have investigated the effects of single use and disposable catheters is limited. Our initial sample size calculation was based on data from a Cochrane review by Prieto et al. (2015), which was later found to contain inaccuracies in

data selection and extraction (9). Specifically, six of the eight trials included in the review did not contain consistent or accurate data compared to their originally published versions. Unfortunately, these erroneous data were used as the basis for our initial sample size calculation (n=456). We were therefore required to perform a new sample size calculation based on the revised 2021 Cochrane review with corrected data (14). The revised Cochrane review included ten studies comparing single-use catheters to reusable catheters. To prevent confounding effects related to insertion techniques (clean vs. sterile), six of these studies employed the same insertions method in both study arms. Among these, three studies provided usable data regarding the number of sUTI events in each group. For single use 18 events out of 68 were observed, for reusable 20 events out of 69. This leads to the proportions of 0.26 and 0.29. Further we applied a power of 0.80 and a one-sided alpha of 0.025 (it is customary to adjust one-sided alphas to the half of 0.05). In our previous calculation, the non-inferiority margin had been determined based on 50% of the mean proportions, following the recommendation of Althunian et al. (15) However, with the corrected data, this would have resulted in an increase in the non-inferiority margin to 14%. As this was deemed inappropriate, we opted to maintain the margin at 11%. The sample size is then calculated with: $n=((Z(1-a)+Z(1-ß))^2 [ps (1-ps)+pe (1-pe)])/((ps-pe-d)^2)$, the formula developed by Blackwelder et al in 1982 (16), leading to 154 effective cases in each group. Anticipating a dropout of 20% (17), this must be divided by 80% and rounded upwards. This results in 2 times 193 participants, a total of 386.

Because the lack of comparable non-inferiority designed trials with the same primary outcome measurement (sUTI) it is chosen to look at non-inferiority trials with a primary outcome measurement of (treatment of) sUTI. All these trials handled a non-inferiority marge of 10% (18) (19-22), and two trials even 15% (23, 24). The head researchers and clinicians of the departments of urology and medical microbiology agreed on the 11% marge to be clinical acceptable.

In addition, we encountered the unique challenge of conducting a study with a significantly extended follow-up period of one year, in contrast to the previous studies used in the sample size calculation above, with a shorter follow-up of 2-4 months. Recognizing the impact of time on the occurrence of urinary tract infections (UTIs) and the need to account for this extended observation period, we also performed a sample size calculation that considered the monthly UTI rates observed by Vapnek et al. (25) This calculation factored in not only the proportion of UTIs but also the temporal aspect, recognizing that the incidence of UTIs may vary over time. Vapnek et al. found that single-use catheters had

an average of 0.13 UTIs per month (SD 0.18) and multiple-use catheters had an average of 0.14 UTIs per month (SD 0.21) per patient (25). We applied the same power of 0.80 and an one-sided alpha of 0.025. A non-inferiority margin of 7% has been determined based on 50% of the means following the recommendation of Althunian et al. (15) We used the most conservative standard deviation of 0.21 and the sample size calculation was performed using the formula developed by Julious: $n = f(\alpha, \beta) \times 2 \times \sigma^2 / d^2$ (26). This calculation led to a requirement of 152 effective cases in each group. Anticipating a dropout rate of 20%, this figure was divided by 0.80, resulting in a total sample size of 382 participants (191 in each group). Since the sample size of 382 is almost equal to our sample size that does not correct for time (n=386), we will include 386 participants to account for both methods.

Sample size: 386 patients.

5. TREATMENT OF SUBJECTS

5.1 Investigational product

The reusable catheter that will be used in this trial will be obtained via Create Medic (http://www.createmedic.co.jp/english/), a Japanese medical manufacturer with broad experience on urological products. The reusable Cliny catheter (http://www.createmedic.co.jp/files/topics/412_ext_11_0.pdf) made by this CE-certified manufacturer, is widely used in Australia and has been shown to be save. Because the catheter manufacturer is CE-certificated and the reusable catheter is considered to be a Class I healthcare device, it does not need a separate CE certificate from a notified body. The reusable catheter is registered in the Z-index and will be covered by the health insurances in the Netherlands. The reusable catheter can be introduced without lubricant because of a high quality smooth surface and will be stored in a holder containing Milton solution, a sterilizing fluid produced by Procter and Gamble which will be renewed every 24 hours. In this trial, the reusable catheters will be used for a period of two weeks, according to the manual of the Royal Children's Hospital Melbourne (https://bit.ly/2tSsj9B). Patients will be trained in using these catheters by continence nurses and will be provided a clear instruction on how to clean and store the catheters.

The control group will be using the single use catheters. These catheters used for intermittent catheterization, are disposable and already reimbursed by standard health insurance in the Netherlands. The choice of the single use catheter will be determined by the preference of the patient.

5.2 Use of co-intervention

Not applicable

5.3 Escape medication

Not applicable

6. INVESTIGATIONAL PRODUCT

6.1 Name and description of investigational product

The Cliny catheter is a catheter that is developed for reuse. The manufacturer, Create Medic, states that the catheter can be safely used for 30 days. In this trial a time period of two weeks for reuse is chosen, according to a manual of the Royal Children's Hospital in Melbourne.

6.2 Summary of findings from non-clinical studies

One non-clinical study is performed by Chan et al, who set up a trial to determine the efficacy of catheter sanitizing and storage for reuse. First catheters were inoculated with e. coli and after washed with antibacterial soap and microwaved or packaged and stored for period of 1, 3 and 7 days. Overall 44% of the catheters washed yielded e. coli vs 26% of the combined washed and microwave treatment, concluding that this is not an absolute way to sanitize catheters for intermittent catheterization (27).

6.3 Summary of findings from clinical studies

There is no literature available for the specific catheter used in this trial, but only of reuse of catheters in general.

A recent systematic review of Prieto et al on intermittent catheterization discussed the scarce evidence in the literature for the differences in safety and efficacy between single-use versus reusable catheters (9). This review concludes that the available data on CIC does not provide convincing evidence that single-use or reuse of catheters is a better strategy. The available trials evaluated were methodologically weak, were generally underpowered, outcome variables differed and follow up differed from up to 24 hours to 12 months. This reflects lack of reliable evidence, rather than evidence of no difference. This Cochrane review was forced to withdrawn in august 2017 with the argument that more fundamental research was necessary to obtain high level evidence (10). This opposing article (10) did an independent appraisal of the data and analyses present in the Cochrane review. In contrast to Prieto et al (9), their analyses revealed a trend to favour single over reusable catheters in respect to the main outcome of UTI.

Kovindha et al describe their cross sectional study on spinal cord injured men in Thailand who had uses CIC with an reusable catheter for over a year. An average time of reuse for each catheter was three years. After two years of reuse, electroscopic findings were encrustation of the catheter, but no obstruction in the lumens and 20% increase in

stiffness. They concluded that reuse of a silicone self-catheter is safe and acceptable for SCI men in development countries, due to low cost and ease of use. To reduce infections induced by catheterization, a shorter period of reuse is recommended and cleansing and disinfection of the catheter should be properly done (8).

Getliff et al performed an earlier systematic review in 2007 and summarizes the evidence on the relationship between sterile single-use catheters or clean reused catheters and the incidence of urinary tract infections (UTIs). After investigation of the 13 trials that met the inclusion criteria, it was stated that there were no definitive studies that illustrated the incidence of UTIs is affected by sterile single-use or coated catheters compared to clean reused catheters. This systematic review concluded that based on the current data, it was not possible to state that one catheter method is better than another and further research on the topic was strongly recommended (28).

Håkansson performed a narrative review in 2014 to summarize all relevant parts needed to make an informed decision whether to choose for single use of reusable catheters in CIC. All relevant found publications were screened and an overall conclusion was formed. The main conclusion of this narrative review was once again that further clinical research is needed to verify/reject safety differences between reuse/single-use catheters (12).

Conclusion of the available literature:
There are no randomized trials available in literature that compare single use and reusable catheters in patients who CIC. Furthermore, the available systematic reviews present conflicting conclusions on the basis of the same studies. Most articles note this lack of knowledge.

6.4 Summary of known and potential risks and benefits

The possible disadvantages of reusable catheters are increased risk of UTIs, effectiveness of cleaning method, damage to the urethra and increased patient burden because of the extra time spend on cleaning the catheter. The possible advantages or benefits of reusable catheters include more patient choice, cost saving, reducing fear of running out of catheters, and environmental advantages.

6.5 Description and justification of route of administration and dosage

All patients will CIC 4-6 times a day, depending on their needs, this will not differ between the intervention and the control group. Patients in the intervention group will use the

reusable catheter and the control group will remain using the single use catheter. All patients are able to catheterize themselves through the urethra.
The reusable catheters are fabricated by Create Medic, a Japanese medical manufacturer with a broad experience in urological products. The reusable catheters are made by the CE-marked manufacturer and registered in the Z-index, so it will be reimbursed through the Dutch health insurances. An independent retailer will provide the catheters to the suppliers in the Netherlands. When a patient decides to withdraw from this trial, they can return the given catheters to the supplier.

6.6 Dosages, dosage modifications and method of administration

Not applicable.

6.7 Preparation and labelling of Investigational Medicinal Product

Not applicable.

6.8 Drug accountability

Not applicable.

7. NON-INVESTIGATIONAL PRODUCT

7.1 Name and description of non-investigational product(s)

Not applicable

7.2 Summary of findings from non-clinical studies

Not applicable

7.3 Summary of findings from clinical studies

Not applicable

7.4 Summary of known and potential risks and benefits

Not applicable

7.5 Description and justification of route of administration and dosage

Not applicable

7.6 Dosages, dosage modifications and method of administration

Not applicable

7.7 Preparation and labelling of Non Investigational Medicinal Product

Not applicable

7.8 Drug accountability

Not applicable

8. METHODS

8.1 Study parameters/endpoints

8.1.1 Main study parameter/endpoint

The main outcome parameters are symptomatic urinary tract infection (sUTI) and hospital admission due to this sUTI. The definition of a sUTI used for this trial is based on the criteria of Woodford et al (29), on the basis of the EAU guidelines on Neurourology (3) and on the basis of the NHG Guidelines for general practitioners (27).

1. Symptomatic UTI (sUTI): A patient with an acute onset of one or more of the following symptoms: dysuria/pain during catheterization, hematuria, frequency, urgency, urinary retention, suprapubic pain, flank pain, fever, delirium or rigors who did not have a negative urine culture result or a negative nitrite test or a negative dipslide/urine sediment (when taken before receiving antibiotics) or a positive blood culture for a known uropathogen. Additionally, in patients with neurogenic bladder a change in specific symptoms, like increase in incontinence, limb spasm and autonomic dysregulation, could be indicative for a sUTI. The diagnosis is to be decided by the local consultant involved in study.

2. Bacteremic UTI (bUTI): A patient with a blood culture positive for a known uropathogen, providing that their urine culture was not negative (when taken before receiving antibiotics).

8.1.2 Secondary study parameters/endpoints

Secondary outcome measurements are symptom specific and quality of life related questionnaires (PGI-I, EQ-5D-5L and SF-Qualiveen), the amount of urethral damage/strictures and kidney/bladder stone formation. To evaluate if urinary cultures/urinary microbiome changes if a patients is reusing a catheter, the patients will hand in catheterized urinary samples for testing at the two clinical visits. Furthermore, economical evaluation of the cost-effectiveness of single-use versus reusable catheters will be performed in cooperation with the health economist within our project group, using the iMCQ and iPCQ questionnaires, which will be entered at week 6, week 26 and week 52. The costs of the Cliny catheter set, including the sterilizing solution, will be included in the economical evaluation. Patients perspective on ease of use and cleaning of the reusable catheters compared to the single-use catheters will be evaluated by the ISC-Q, the InCaSaQ and, for the

intervention group, also the PGI-I questionnaire. These questionnaires are developed to investigate difficulties in use, the impact of catheterization on patients and the overall acceptance in patients and will be entered at baseline, week 6, week 26 and week 52. Overall quality of life will be evaluated by the EQ-5D-5L measure method and the SF-Qualiveen and will be entered at baseline, week 6, week 26 and week 52. All used questionnaires are validated in Dutch.

Two additional questions concerning patients thoughts on environmental burden and healthcare costs will be asked at week 0 and week 52.

8.1.3 Other study parameters

Other parameters such as patients characteristics, underlying (immune)diseases, hand function and mobility will be assessed.

8.2 Randomisation, blinding and treatment allocation

Patients who sign informed consent will be randomised into the intervention or in the control group. Randomisation will be performed using the program ALEA, which is the program used in the Erasmus MC. ALEA is developed for randomisation and guarantees concealed allocation. The groups will be stratified for the participating centres, neurogenic and non-neurogenic causes for catheterization, age (16-17 years vs. ≥ 18 years and < 50 years vs. ≥ 50 years old), gender, and the female patient group will be balanced for pre- and post-menopausal status. There is no ability for blinding in this trial.

8.3 Study procedures

This study will be a prospective non-inferiority trial with two randomized groups. The patients will be screened, included and randomized at the outpatient clinic of the participating hospital departments. One group of patients will use the reusable catheter and the control group will use the single use catheters. After inclusion, the patients will be followed up for a period of twelve months. See chapter 3 'Study design' for the precise follow up schedule.

There will be two extra clinical consults and seventelephone consults when participating in this study. Patients will receive a financial compensation for the expenses of traveling to the hospital for the clinical consultations.

Tracking UTIs and hospital admissions will be performed by status analysis by the researchers of the trial and patients will track their infections using a diary. Active cooperation of the involved consultants and continence nurses is asked when the participating centers are included in the trial. For the contact moments, standardized

questions will be provided in Gemstracker, so all patients will be asked the same questions and data will be as complete and similar as possible.

8.4 Withdrawal of individual subjects

Subjects can leave the study at any time for any reason if they wish to do so without any consequences. The investigator can decide to withdraw a subject from the study for urgent medical reasons.

8.4.1 Specific criteria for withdrawal

When it turns out during the trial that a patient is not able to apply the cleaning method the correct way, they will be offered a new schooling moment with a continence nurse. When an incorrect cleaning method is persistent, they will be excluded from the trial, to protect patients' safety.

8.5 Replacement of individual subjects after withdrawal

There will be no replacement after withdrawal.

8.6 Follow-up of subjects withdrawn from treatment

When a patient is withdrawn from the trial, regardless of the reason for withdrawal, follow up will be terminated because patients will return to their original single use regime.

8.7 Premature termination of the study

When the trial shows a substantial amount of serious adverse events in the intervention group and patients' safety is questioned, the principal investigator can decide to terminate the study prematurely. All participants will then return to the single use regime.

9. SAFETY REPORTING

9.1 Temporary halt for reasons of subject safety

In accordance to section 10, subsection 4, of the WMO, the sponsor will suspend the study if there is sufficient ground that continuation of the study will jeopardise subject health or safety. The sponsor will notify the accredited METC without undue delay of a temporary halt including the reason for such an action. The study will be suspended pending a further positive decision by the accredited METC. The investigator will take care that all subjects are kept informed.

9.2 AEs, SAEs and SUSARs

9.2.1 Adverse events (AEs)

Adverse events are defined as any undesirable experience occurring to a subject during the study, whether or not considered related to the investigational product. All adverse events reported spontaneously by the subject or observed by the investigator or his staff will be recorded.

9.2.2 Serious adverse events (SAEs)

A serious adverse event is any untoward medical occurrence or effect that

- results in death;
- is life threatening (at the time of the event);
- requires hospitalisation or prolongation of existing inpatients' hospitalisation;
- results in persistent or significant disability or incapacity;
- is a congenital anomaly or birth defect; or
- any other important medical event that did not result in any of the outcomes listed above due to medical or surgical intervention but could have been based upon appropriate judgement by the investigator.

An elective hospital admission will not be considered as a serious adverse event.

The only expected SAE that may occur is a urosepsis after a sUTI is not recognized or acknowledged, which can occur in both the intervention and the control group.

The following situations are not considered to be SAEs and should not be reported:

- elective hospitalisation for pre-existing conditions that have not been exacerbated by trial participation.
- hospitalisation which was planned before the patient consented for study participation and where admission did not take longer than anticipated.
- medical or surgical procedure (e.g. endoscopy, appendectomy); the condition that leads to the procedure is an (S)AE, unless the condition pre-existed before trial participation.
- situations where an untoward medical occurrence did not occur (social and/or convenience admission to a hospital, palliative care, rehabilitation, overdose without occurrence of an adverse event).
- anticipated day-to-day fluctuations of pre-existing disease(s) or condition(s) present or detected at the start of the study that do not worsen.
- events that are considered to be directly linked to the standard procedures.
- events that are considered not to be linked to any study procedure.

The investigator will report all SAEs to the sponsor without undue delay after obtaining knowledge of the events.

The sponsor will report the SAEs through the web portal *ToetsingOnline* to the accredited METC that approved the protocol, within 7 days of first knowledge for SAEs that result in death or are life threatening followed by a period of maximum of 8 days to complete the initial preliminary report. All other SAEs will be reported within a period of maximum 15 days after the sponsor has first knowledge of the serious adverse events.

9.2.3 Suspected unexpected serious adverse reactions (SUSARs)

Not applicable

9.3 Annual safety report

In addition to the expedited reporting of SUSARs, the sponsor will submit, once a year throughout the clinical trial, a safety report to the accredited METC, competent authority, and competent authorities of the concerned Member States. This safety report consists of:

- a list of all suspected (unexpected or expected) serious adverse reactions, along with an aggregated summary table of all reported serious adverse reactions, ordered by organ system, per study;

- a report concerning the safety of the subjects, consisting of a complete safety analysis and an evaluation of the balance between the efficacy and the harmfulness of the medicine under investigation.

9.4 Follow-up of adverse events

All AEs will be followed until they have abated, or until a stable situation has been reached. Depending on the event, follow up may require additional tests or medical procedures as indicated, and/or referral to the general physician or a medical specialist. SAEs need to be reported till end of study within the Netherlands, as defined in the protocol

9.5 Data Safety Monitoring Board (DSMB) / Safety Committee

Not applicable.

10. STATISTICAL ANALYSIS

10.1 Primary study parameters

For analysis of the results, the groups will be stratified for gender and de female patient group will be balanced for pre- and post-menopausal. Data analysis will be performed using SPSS. The primary analysis will be to assess difference between the intervention and the control groups in the sUTI rate and other adverse effects. Descriptive statistics will be used to describe baseline characteristics of participating patients in both groups. Binomial of categorical outcome measures will be analyzed using Chi-square tests and quantitative outcome variables by t-tests or Mann-Whitney U tests. Two-sides p values are calculated.

10.2 Secondary study parameters

Secondary outcome measurements are symptom specific and quality of life related questionnaires (PGI-I, SF-Qualiveen, EQ-5D-5L), the amount of urethral damage/strictures, kidney/bladder stone formation and changes in urinary culture/urinary microbiome. The ease use of the catheters and patients satisfaction will be evaluated by the ISC-Q and the InCaSaQ.

Cost-effectiveness: For the purpose of assessing the cost-effectiveness of reusable catheters data will be collected on medical healthcare utilization, productivity losses and quality of life of patients alongside the clinical trial. In this cost-effectiveness study, incremental costs and incremental effects of reusable catheters over single-use catheters will be assessed, with effects expressed in quality adjusted life-years (QALYs). The cost-effectiveness study will adhere to the Dutch health economic guidelines. As such the societal perspective will be adopted, meaning that all costs and effects will be included in the analyses, regardless to whom they accrue. The time horizon of the cost-effectiveness study will be equal to the timeframe of the clinical trial.
Uncertainty concerning the ICER, QALYs and costs will be assessed using bootstrapping, and this uncertainty will be presented graphically with the CE- acceptability curve.
Data on medical healthcare utilization (i.e. volumes) will be collected both through the hospital and by means of the iMTA Medical Consumption Questionnaire (iMCQ). The iMCQ is a generic, non-disease specific, questionnaire to be filled out by the patient, and assesses the use of a variety of medical services. Hospital records can be used to obtain information about consultations and admissions for symptomatic UTIs.
Costs of catheters will be derived from the hospital financial administration. For single-use catheters, this is equal to the purchase price of the catheters. For reusable catheters,

costs of e.g. cleaning also need to be taken into account. This will be based on the time it takes plus possibly material to prepare a reusable catheter to be used again. This information will be estimates based on e.g. observations and/or expert opinion.
The data collected on healthcare utilization will be multiplied by standard unit cost prices, which are described in the Dutch costing manual.
Data on productivity losses will be collected by means of the iMTA Productivity Costs Questionnaire (iPCQ). The iPCQ is a generic, non-disease specific, questionnaire. The iPCQ assesses productivity losses from absence from work or reduced efficiency at work and unpaid work. The data collected from the iPCQ will be monetized with hourly productivity costs derived from the Dutch costing manual. The five level version of the Euroqol 5D (EQ-5D-5L) will be used to measure quality of life of patients. The questionnaire consists of five dimensions, each with five answer levels. For cost-effectiveness studies, quality of life is often expressed in utilities, which present quality of life on a scale from 0 (death) to 1 (perfect health). The EQ-5D-5L can be used to calculated utilities using a Dutch tariff. QALYs are calculated by combining quality of life and survival. The EQ-5D-5L questionnaire will be administered at time 0, 6, 26 weeks and 52 weeks, whilst the iMCQ and iPCQ will be administered at time 6, 26 weeks and 52 weeks.

10.3 Other study parameters

Other parameters such as patients characteristics, underlying (immune)diseases, hand function and mobility will be assessed with descriptive statistics.

10.4 Interim analysis

Not applicable

11. ETHICAL CONSIDERATIONS

11.1 Regulation statement

The study will be conducted to the standards of Good Clinical Practice, in full conformance with the "Declaration of Helsinki" (version October 2013), the Dutch laws and regulations, with the WMO ("Wet Medisch-wetenschappelijk Onderzoek met mensen") in particular.

11.2 Recruitment and consent

All eligible patients will be asked to participate in this trial by their treating physician at the urological outpatients clinic of the participating centres. After a patient is well informed by the physician, a one-week period to consider his/her decision will be facilitated. After one week the researchers will discuss the decision of the patient by telephone and will answer any risen questions. When a patient decides to participate, the appointment of the first clinical visit will be planned. Signing the informed consent and randomization will be performed during this visit.

11.3 Objection by minors or incapacitated subjects

Not applicable.

11.4 Benefits and risks assessment, group relatedness

The possible risks of reusable catheters are increased risk of UTIs, which is the main outcome measurement of this trial. These risks may occur more often when the cleaning method is not effectively performed, which will be evaluated thoroughly during the trial. Other risks may be damage to the urethra and increased patient burden because of the cleaning procedure of the catheter. The possible advantages or benefits of reusable catheters include more patient choice, reducing fear of running out of catheters, cost saving and environmental advantages.

11.5 Compensation for injury

The participating centres have a liability insurance which is in accordance with article 7 of the WMO.

The sponsor has an insurance which is in accordance with the legal requirements in the Netherlands (Article 7 WMO). This insurance provides cover for damage to research subjects through injury or death caused by the study.

The insurance applies to the damage that becomes apparent during the study or within 4 years after the end of the study.

11.6 Incentives

Patients will receive a compensation for their expenses for the three or four extra consultations in the hospital. This will be 20 Euro for each consultation.

12. ADMINISTRATIVE ASPECTS, MONITORING AND PUBLICATION

12.1 Handling and storage of data and documents

All data will be handled confidentially and will be coded. A subject identification code list will be used to link the data to the subject. The code will not be based on the patients` initials and/or birth-date. Only the researchers involved in this trial will have the key to link the patients included in their center to their data. Handling of data will comply with the EU General Data Protection Regulation and the Dutch Act on Implementation of the General Data Protection Regulation.

12.2 Monitoring and Quality Assurance

Monitoring will be done following the requirements of the Netherlands Federation of University Medical Centres (NFU) based on the ICH Good Clinical Practice guidelines. Monitoring will be carried out by qualified monitors of the Trialbureau Erasmus MC and dedicated urology research nurses. The frequency of complications due to participation in this trial are expected to be low and of low severity and not more often or severe than in the normal population. Therefore, the investigators propose to classify this study as a low-risk study. For a detailed risk and benefit assessment, please see paragraph 11.3. For low-risk clinical trials monitoring will comprise one visit per study site per year. A detailed data monitoring plan will be set up following the standardized requirements of ZonMw.

12.3 Amendments

Amendments are changes made to the research after a favourable opinion by the accredited METC has been given. All amendments will be notified to the METC that gave a favourable opinion.

12.4 Annual progress report

The sponsor/investigator will submit a summary of the progress of the trial to the accredited METC once a year. Information will be provided on the date of inclusion of the first subject, numbers of subjects included and numbers of subjects that have completed the trial, serious adverse events/ serious adverse reactions, other problems, and amendments.

12.5 Temporary halt and (prematurely) end of study report

The investigator/sponsor will notify the accredited METC of the end of the study within a period of 8 weeks. The end of the study is defined as the last patient’s last visit.

The sponsor will notify the METC immediately of a temporary halt of the study, including the reason of such an action.
In case the study is ended prematurely, the sponsor will notify the accredited METC within 15 days, including the reasons for the premature termination.

Within one year after the end of the study, the investigator/sponsor will submit a final study report with the results of the study, including any publications/abstracts of the study, to the accredited METC.

12.6 Public disclosure and publication policy

Data collected in this trial will be disclosed to the public through peer-reviewed publications and presentations.

13. STRUCTURED RISK ANALYSIS

13.1 Potential issues of concern

Not applicable.

a. Level of knowledge about mechanism of action

b. Previous exposure of human beings with the test product(s) and/or products with a similar biological mechanism

c. Can the primary or secondary mechanism be induced in animals and/or in *ex-vivo* human cell material?

d. Selectivity of the mechanism to target tissue in animals and/or human beings

e. Analysis of potential effect

f. Pharmacokinetic considerations

g. Study population

h. Interaction with other products

i. Predictability of effect

j. Can effects be managed?

13.2 Synthesis

The risks that may occur during this trial are risks that are associated with CIC, the aim of this trial is to assess if these risks occur more often when a catheter is reused. Patients will be well trained in the cleaning method, to keep this risk as low as possible. Patients will be informed when to consult a physician. Most patients will have a lot of experience because they are performing CIC for a long period of time already. Patients who are starting CIC will have a learning time of two weeks before they are included in the trial and randomized, so good practice is assured.

If the cleaning method is not accurately performed after extra schooling and there is a high change of infections, patients will be withdrawn from the trial.

14. REFERENCES

1. Chancellor MB, Diokno AC, (Eds). The Underactive Bladder. Spinger International Publishing. 2016.
2. Groen J, Pannek J, Castro Diaz D, Del Popolo G, Gross T, Hamid R, et al. Summary of European Association of Urology (EAU) Guidelines on Neuro-Urology. Eur Urol. 2016;69(2):324-33.
3. B. Blok (Co-chair) JPC-c, D. Castro-Diaz, G. del Popolo, J. Groen, R. Hamid, G. Karsenty, T.M. Kessler, Guidelines Associates: H. Ecclestone BP-F, L. 't Hoen, S. Musco, V. Phé, S. Reuvers, M.P. Schneider. EAU guideline on Neuro-urology. Retrieved from http://uroweborg/guideline/neuro-urology/ Access date 22-01-2018. 2017.
4. Urinary Catheters Market Analysis by Product, (Intermittent Catheters, Foley/Indwelling Catheters, External Catheters), By Application, (Benign Prostate Hyperplasia, Urinary Incontinence, Others), And Segment Forecasts, 2013 - 2024. wwwgrandviewresearchcom. 2017.
5. Vahr S, Cobussen-Boekhorst H, Eikenboom J, Geng V, Holroyd S, Lester M, et al. EAUN Evidence-based Guideline for: Best Practice in Urological Health Care - Catheterisation - Urethral intermittent in adults. http://wwwuroweborg/nurses/nursingguidelines/. March 2013.
6. Richtlijn Blaaskatheters - Langdurige blaaskatheterisatie bij patienten met complexe multimorbiditeit. Verenso, versie
april 2011.
(http://www.verenso.nl/assets/Uploads/Downloads/Richtlijnen/VerensoRichtlijnblaaskatheters2.pdf).
7. Bogaert GA, Goeman L, de Ridder D, Wevers M, Ivens J, Schuermans A. The physical and antimicrobial effects of microwave heating and alcohol immersion on catheters that are reused for clean intermittent catheterisation. Eur Urol. 2004;46(5):641-6.
8. Kovindha A, Mai WN, Madersbacher H. Reused silicone catheter for clean intermittent catheterization (CIC): is it safe for spinal cord-injured (SCI) men? Spinal Cord. 2004;42(11):638-42.
9. Prieto JA, Murphy C, Moore KN, Fader MJ. Intermittent catheterisation for long-term bladder management (abridged cochrane review). Neurourol Urodyn. 2015;34(7):648-53.
10. Christison K, Walter M, Wyndaele JJM, Kennelly M, Kessler TM, Noonan VK, et al. Intermittent catheterization: The devil is in the details. J Neurotrauma. 2017.
11. McClurg D, Coyle J, Long A, Moore K, Cottenden A, May C, et al. A two phased study on health care professionals' perceptions of single or multi-use of intermittent catheters. Int J Nurs Stud. 2017;72:83-90.
12. Hakansson MA. Reuse versus single-use catheters for intermittent catheterization: what is safe and preferred? Review of current status. Spinal Cord. 2014;52(7):511-6.
13. Sun AJ, Comiter CV, Elliott CS. The cost of a catheter: An environmental perspective on single use clean intermittent catheterization. Neurourol Urodyn. 2018;37(7):2204-8.
14. Prieto JA, Murphy CL, Stewart F, Fader M. Intermittent catheter techniques, strategies and designs for managing long-term bladder conditions. Cochrane Database Syst Rev. 2021;10:CD006008.
15. Althunian TA, de Boer A, Groenwold RHH, Klungel OH. Defining the noninferiority margin and analysing noninferiority: An overview. Br J Clin Pharmacol. 2017;83(8):1636-42.
16. Blackwelder WC. "Proving the null hypothesis" in clinical trials. Control Clin Trials. 1982;3(4):345-53.
17. Cardenas DD, Moore KN, Dannels-McClure A, Scelza WM, Graves DE, Brooks M, et al. Intermittent catheterization with a hydrophilic-coated catheter delays urinary tract infections in acute spinal cord injury: a prospective, randomized, multicenter trial. Pm R. 2011;3(5):408-17.
18. van Nieuwkoop C, van't Wout JW, Assendelft WJ, Elzevier HW, Leyten EM, Koster T, et al. Treatment duration of febrile urinary tract infection (FUTIRST trial): a randomized placebo-controlled multicenter trial comparing short (7 days) antibiotic treatment with conventional treatment (14 days). BMC Infect Dis. 2009;9:131.

19. van Nieuwkoop C, van der Starre WE, Stalenhoef JE, van Aartrijk AM, van der Reijden TJ, Vollaard AM, et al. Treatment duration of febrile urinary tract infection: a pragmatic randomized, double-blind, placebo-controlled non-inferiority trial in men and women. BMC Med. 2017;15(1):70.
20. Wagenlehner FM, Umeh O, Steenbergen J, Yuan G, Darouiche RO. Ceftolozane-tazobactam compared with levofloxacin in the treatment of complicated urinary-tract infections, including pyelonephritis: a randomised, double-blind, phase 3 trial (ASPECT-cUTI). Lancet. 2015;385(9981):1949-56.
21. Vik I, Bollestad M, Grude N, Baerheim A, Damsgaard E, Neumark T, et al. Ibuprofen versus pivmecillinam for uncomplicated urinary tract infection in women-A double-blind, randomized non-inferiority trial. PLoS Med. 2018;15(5):e1002569.
22. Ten Doesschate T, van Mens SP, van Nieuwkoop C, Geerlings SE, Hoepelman AIM, Bonten MJM. Oral fosfomycin versus ciprofloxacin in women with E.coli febrile urinary tract infection, a double-blind placebo-controlled randomized controlled non-inferiority trial (FORECAST). BMC Infect Dis. 2018;18(1):626.
23. Wagenlehner FM, Abramov-Sommariva D, Holler M, Steindl H, Naber KG. Non-Antibiotic Herbal Therapy (BNO 1045) versus Antibiotic Therapy (Fosfomycin Trometamol) for the Treatment of Acute Lower Uncomplicated Urinary Tract Infections in Women: A Double-Blind, Parallel-Group, Randomized, Multicentre, Non-Inferiority Phase III Trial. Urol Int. 2018;101(3):327-36.
24. Ren H, Li X, Ni ZH, Niu JY, Cao B, Xu J, et al. Treatment of complicated urinary tract infection and acute pyelonephritis by short-course intravenous levofloxacin (750 mg/day) or conventional intravenous/oral levofloxacin (500 mg/day): prospective, open-label, randomized, controlled, multicenter, non-inferiority clinical trial. Int Urol Nephrol. 2017;49(3):499-507.
25. Vapnek JM, Maynard FM, Kim J. A prospective randomized trial of the LoFric hydrophilic coated catheter versus conventional plastic catheter for clean intermittent catheterization. J Urol. 2003;169(3):994-8.
26. Julious SA. Sample sizes for clinical trials with normal data. Stat Med. 2004;23(12):1921-86.
27. Chan JL, Cooney TE, Schober JM. Adequacy of sanitization and storage of catheters for intermittent use after washing and microwave sterilization. J Urol. 2009;182(4 Suppl):2085-9.
28. Getliffe K, Fader M, Allen C, Pinar K, Moore KN. Current evidence on intermittent catheterization: sterile single-use catheters or clean reused catheters and the incidence of UTI. J Wound Ostomy Continence Nurs. 2007;34(3):289-96.
29. Woodford HJ, George J. Diagnosis and management of urinary tract infection in hospitalized older people. J Am Geriatr Soc. 2009;57(1):107-14.
27. Van Pinxteren B KB, Geerlings SE, Visser HS, Klinkhamer S, Van der Weele GM, Verduijn MM, Opstelten W, Burgers JS, Van Asselt KM. NHG-Standaard Urineweginfecties(derde herziening) Huisarts Wet. 2013;56(6):270-80.

STATISTICAL ANALYSIS PLAN for COMPaRE-trial

Administrative information:

Sponsor name	Department of Urology, Erasmus MC, The Netherlands
Sponsor address	Dr. Molewaterplein 40, 3015GD, Rotterdam
EU CT number / REC no	Not applicable
Trial title	Single use vs reusable catheters in intermittent CatheterizatiOn for treatment of urinary retention: a Multicenter, Prospective, RandomizEd controlled, non-inferiority trial (COMPaRE)
Trial ID	COMPaRE-trial
Trial registration number	NL-OMON54700<br>(International Clinical Trials Registry Platform: NL8296)

SAP and protocol version:

SAP version and date:	SAP V1.0, 27-10-2025
Protocol version	This document has been written based on information contained in the study protocol version 5.0, dated 03-10-2023

SIGNATURE PAGE

PRINCIPAL/COORDINATING INVESTIGATOR:

Prof. Bertil F.M. Blok
Dept. Urology, Erasmus University Medical Center,
Rotterdam.

27-10-2025

Signature

Date (dd/mmm/yyyy)

TRIAL STATISTICIAN:

Prof. Bettina Hansen
Dept. Department of Epidemiology and Biostatistics,
Erasmus University Medical Center, Rotterdam.

11-11-2025

Signature

Date (dd/mmm/yyyy)

ABBREVIATIONS

AE	Adverse Event
BMI	Body Mass Index
CE	Conformité Européenne
CEA	Cost Effectiveness Analysis
CI	Confidence Interval
CI(S)C	Clean Intermittent (Self) Catheterization
COPD	Chronic Obstructive Pulmonary Disease
DM2	Diabetes Mellitus type 2
EAU	European Association of Urology
FAS	Full Analysis Set
ITT	Intention to treat
KC	Clinical Consult
MAR	Missing at Random
NA	Not applicable
NHG	Nederlandse Huisartsen Gemeenschap (Dutch general practitioner community)
NLUTD	Neurogenic Lower Urinary Tract Dysfunction
PP	Per Protocol
PROM	Patient Reported Outcome Measure
QALY	Quality Adjusted Life Years
QoL	Quality of Life
SAE	Serious Adverse Event
SD	Standard Deviation
SOC	System Organ Class
sUTI	systemic Urinary Tract Infection

TC	Telephone Consult
UTI	Urinary Tract Infection
VAS	Visual Analog Scale

TABLE OF CONTENTS

1 Table of Contents

2 INTRODUCTION 7
2.1 Background and Rationale 7
2.2 Intervention(s) 7
2.2.1 Brief description of the study intervention(s) 7
2.2.2 Control settings (if applicable) 7
2.3 Trial Objectives 7
1.3.1 Primary Objective 7
2.3.2 Secondary Objectives 7
3 TRIAL METHODS 8
3.1 Trial Design 8
3.2 Randomisation 8
3.3 Statistical Framework 9
3.3.1 Hypothesis Test 9
3.3.2 Confidence Intervals and p-values 9
3.3.3 Decision Rule 9
3.4 Timing of Outcome Assessments 9
3.5 Statistical Interim Analyses and Stopping Guidance 10
3.6 Timing of Main Analysis 10
4 TRIAL POPULATION 10
4.1 Screening Data, Eligibility and Recruitment 10
4.2 Baseline Patient Characteristics 10
4.3 Withdrawal/Follow-up 11
4.4 Adherence and Protocol Deviations 11
4.4.1 Adherence to Allocated Treatment 11
4.4.2 Protocol Deviations 11
4.5 Analysis Populations 12
5 OUTCOME DEFINITIONS 12
5.1 General Definitions and Derived Variables 12
5.1.1 Body Mass Index (BMI) 12
5.1.2 Urine cultures 12
5.1.3 Other calculations 12
5.2 Primary Outcome Definition 12
5.3 Secondary Outcomes Definitions 13
5.3.1 Hospitalisation due to UTI 13
5.3.2 Bacteraemic UTI/ urosepsis 13
5.3.3 Urethral stricture 13
5.3.4 Kidney/bladder stone formation 13

5.3.5 Episode of macroscopic hematuria 13
5.3.6 Patient satisfaction 13
5.3.7 Quality of life (QoL) 14
5.3.8 Cost-effectiveness 14
5.3.9 Patient opinion 15
5.3.10 Changes in urine cultures over time 15
5.4 Overview of Outcomes 15
6 ANALYSIS METHODS 16
6.1 Methods for Primary Outcome 16
6.1.1 Descriptive Statistics 16
6.1.2 Primary Analysis 17
6.1.3 Missing Data 17
6.1.4 Subgroup Analyses .... **Error! Bookmark not defined.**
6.2 Methods for secondary outcomes 17
6.2.1 Methods (S)AE Secondary Outcomes 17
6.2.2 Methods for outcomes of questionnaires (ISCQ, InCaSaQ, EQ-5D-5L, SF-Qualiveen, PGI-I). 17
6.2.3 Methods for the outcome of Patient opinion questionnaire 18
6.2.4 Methods for changes in urine culture 18
6.3 Sample size 18
7 SAFETY ANALYSES 19
7.1 Adverse Events 19
7.2 Clinical Laboratory Parameters 19
7.3 Vital Signs 20
8 STATISTICAL SOFTWARE 20
9 REFERENCES 20
9.1 Literature References 20

2 Introduction

2.1 Background and Rationale

Urinary retention affects millions of people worldwide and clean intermittent self-catheterization (CISC) is the preferred treatment. In many high-income countries, single-use plastic catheters is the standard for CISC, with the average individual patient using four catheters daily resulting in approximately 1,825 catheters per year. In the U.S. alone, this generates around 206 million liters of waste annually - equivalent to more than 80 Olympic-size swimming pools. With growing concerns about environmental sustainability and rising healthcare costs, reusable catheters have emerged as a potential alternative. However, the safety and efficacy of reusable catheters, particularly regarding urinary tract infections (UTIs), remain uncertain due to limited quality and conflicting evidence.

This document outlines the planned data summaries and statistical analyses to be performed for the COMPaRE trial: A multicenter, prospective, randomized, non-inferiority study designed to evaluate whether reusable catheters are as safe as single-use catheters for intermittent catheterization in patients with urinary retention. The information presented here is intended to supplement the study protocol, which contains details regarding the objectives and design of the study.

2.2 Intervention(s)

2.2.1 Brief description of the study intervention(s)

Patients in the intervention arm will receive instructions and start using the Cliny catheter (males and females) or the PureCath catheter (only females). These reusable catheters can be introduced without lubricant because of a high-quality smooth surface and will be stored in a holder containing a diluted 2% sodium hypochlorite solution, which will be renewed every 24 hours. The 2% sodium hypochlorite solution is diluted with cold tap water (1:80). In this trial, Milton fluid (a product of Procter and Gamble) is used to clean and store the catheter. To reduce the risk of damage from the cleaning solution, the catheter is rinsed with cold tap water before and after each use. Every reusable catheter will be used for 2 weeks. The reusable catheters are Conformité Européenne (CE)-marked which indicates that the manufacturer confirms the product’s compliance with European Union legislation for medical devices (Regulation 2017/745). The manufacturer of the reusable catheter (Create Medic) tested the compatibility of cleaning solution with the reusable catheters and recommended the use of 0.6% dilution of 2% sodium hypochlorite w/w solution as cleaning method.

2.2.2 Control settings (if applicable)

Patients allocated to the control arm will remain using their own single-use catheters, the choice of the single-use catheter will b determined by the preference of the patient.

2.3 Trial Objectives

1.3.1 Primary Objective

To determine whether reusable catheters are as safe as single-use catheters, measured by UTI/month. The safety analysis, including the (S)AE’s will also be presented with the primary results.

2.3.2 Secondary Objectives

Our secondary objectives are to investigate the efficiency and costs-effectiveness of the reusable catheter and to explore patient opinions on the reusable catheter. The table below provides an overview of all objectives and outcome measures.

Objectives	Primary outcome	Secondary outcome	Measured by
**Safety**<br>To determine whether reusable catheters are at least not less safe as single use catheters	No of sUTIs	► Hospitalisation due to a sUTI<br>► Bacteraemic UTI<br>► Urethral damage leading to clinical significant strictures<br>► Kidney/bladder stone formation<br>► Episodes of macroscopic haematuria	► sUTI (see definition)<br>► sUTI +hospitalisation records<br>► sUTI +positive blood culture<br>► Anamnestic<br>► Anamnestic<br>► Anamnestic
**Efficiency**<br>To investigate whether reusable catheters are not less efficient as single use catheters	X	► Patient satisfaction<br>► Quality of life	► PROMs: ISCQ, InCaSaQ, PGI-I<br>► PROM: EQ-5D-5L
**Costs-effectiveness**<br>To investigate whether reusable catheters are costs-effective in comparison to single use catheters	X	► Quality-adjusted-life-years and incremental costs-effecitiveness ratios	► Hospital records<br>► PROMs: iMCQ, iPCQ, EQ-5D-5L
**Patient opinions**<br>To explore patients opinions on healthcare costs and environmental burden in the context of CISC	X	► Patient opinion	► Two statement questions answered by a Likert-scale from 1 to 5 (fully agree – fully disagree)

CISC, clean intermittent urinary self-catheterisation; EQ-5D-5L, Euroqol 5 Dimensional 5 Level; iMCQ, iMTA Medical Consumption Questionnaire; InCaSaQ, Intermittent Catheterisation Satisfaction Questionnaire; iPCQ, iMTA Productivity Costs Questionnaire; ISCQ, Intermittent Self-Catheterisation Questionnaire; PGI-I, Patient Global Impression of Improvement; PROMs, patient-reported outcome measurements; sUTI, symptomatic urinary tract infection.

3 Trial Methods

3.1 Trial Design

The COMPaRE trial is a multicentre randomised non-inferiority trial, conducted at the urological department of the Erasmus Medical Center (Erasmus MC). Patients are randomly assigned according to the minimization method to either single-use catheters (control arm) or reusable catheters (intervention arm).

3.2 Randomisation

Randomisation is done by the tool ALEA (meaning 'dice' in Latin), according to the regulations of the Erasmus MC. ALEA is developed for randomisation and guarantees concealed allocation. The intervention and control group will be stratified for the participating centres, neurogenic and non-neurogenic causes for catheterisation, age (16–17 years vs ≥18 years and < 50 years vs. ≥ 50 years old), gender, and the female patient group will be balanced for premenopausal and postmenopausal status. On randomisation, patients will be allocated a unique study subject number in chronologically ascending order for every study site, starting with 1 (e.g., Erasmus MC: EMC001). They will be randomized to the intervention arm (reusable catheter) or control arm (single use catheter). There is no prespecified list on randomisation, but each combination of stratification factors will form a combination. Within each combination, ALEA will randomly assign a study arm. The rational for this approach is that it will maximise the probability of assigning a new participant in

the study arm with the lowest number of patients. The company for the randomisation procedure is the Clinical Trial Centre (CTC) of the Erasmus MC.

3.3 Statistical Framework

3.3.1 Hypothesis Test

This trial is designed to demonstrate the non-inferiority of reusable catheters compared to single-use catheters with regard to the monthly incidence of UTIs over a one-year study period, in patients who perform intermittent self-catheterization due to urinary retention.

- The primary null hypothesis is that reusable catheters are inferior to a single-use catheters with regard to the monthly incidence of UTIs over the study period, with a 7% inferiority margin.
- The alternative null hypothesis is that reusable catheters are non-inferior to a single-use catheters with regard to the monthly incidence of UTIs over the study period, with a 7% inferiority margin.

3.3.2 Confidence Intervals and p-values

All calculated p-values will be two-sided and compared to a 5% significance level. If a p-value is less than 0.05, the corresponding treatment group difference will be denoted as statistically significant. All efficacy estimates will be presented with two-sided 95% confidence intervals. As there is only one primary null hypothesis to be tested in this trial, there will be no adjustments for multiplicity.

3.3.3 Decision Rule

This trial is designed to address a single primary outcome. Non-inferiority is claimed if the primary null hypothesis is rejected on the significance level (alpha) of 0.025 (one-sided). That is, if the upper-limit of the two-sided 95% confidence interval for the treatment difference is less than 7%.

3.4 Timing of Outcome Assessments

For all clinically planned measures, visits should occur within a window of the scheduled visit. The target day and visits window is defined in the protocol as:

**Label**	**Target Week**	**Definition (Week window)**
KC0, baseline (Day 0)	Week 0 (randomisation)	Day 0
TC1	Week 1	Target week +/- 3 weeks
KC2	Week 6	Target week +/- 3 weeks
TC2	Week 12	Target week +/- 3 weeks
TC3	Week 18	Target week +/- 3 weeks
KC3	Week 26	Target week +/- 3 weeks

TC4	Week 34	Target week +/- 3 weeks
TC5	Week 42	Target week +/- 3 weeks
KC4, last study visit*	Week 52	Target week +/- 3 weeks

*The last study visit is defined as the visit following the last visit with randomised treatment, and where there is a study end statement.

3.5 Statistical Interim Analyses and Stopping Guidance

There will be no interim analyses in this trial.

3.6 Timing of Main Analysis

The main analysis is planned when all patients have concluded 52 ± 4 weeks of treatment, all data up to 52 weeks have been entered, verified and validated and the primary database has been locked.

4 Trial Population

4.1 Screening Data, Eligibility and Recruitment

A CONSORT flow diagram (appendix A) will be used to summarise the number of patients who were:

- assessed for eligibility at screening
- eligible at screening
- ineligible at screening*
- eligible and randomised
- received the randomised allocation
- lost to follow-up*
- discontinued the intervention*
- randomised and included in the primary analysis
- randomised and excluded in the primary analysis*

*reasons will be provided.

4.2 Baseline Patient Characteristics

The patient demographics and baseline characteristics to be summarised include age in years, BMI, sex, menopausal status, race, comorbidities (COPD, DM2, hypertension, cardiovascular disease), underlying cause of catheterization (e.g., NLUTD vs non-NLUTD), spontaneous miction, hand function, years of CIC experience, CIC frequency, Antibiotic prophylaxis, bladder irrigation, intradetrusor botulinum toxin A, self-reported UTI in previous 6 months before trial.

Patient demographics and baseline characteristics will be summarised by randomised treatment arm and overall using descriptive statistics (N, mean, standard deviation, median, 25/75 percentiles, minimum, and maximum) for continuous variables, and number and percentages of patients for categorical variables. There will be no statistical analysis of treatment difference. Any clinical important imbalance between the treatment groups will be noted.

4.3 Withdrawal/Follow-up

The status of eligible and randomised patients during the trial will be presented in a flowchart according to treatment group.

- completed intervention
- withdrew consent
- lost to follow-up

Time from randomisation to treatment discontinuation and time from randomisation to withdrawal/lost of follow-up will be presented in a table. Information on the number of withdrawals, reasons for withdrawal, and number included in the analysis will be provided.

4.4 Adherence and Protocol Deviations

4.4.1 Adherence to Allocated Treatment

Adherence is assessed based on the percentage of patients in the intervention arm who use single-use catheters within the acceptable limit. In this study, patients in the intervention arm are allowed to use a maximum of 20% single-use catheters. If the percentage exceeds 20% persistently, the patient will be considered non-compliant.

Patients in the single-use catheter arm are required to use only single-use catheters and may not reuse them.

Data summarizing the proportions of patients complying with the treatment regimen according to protocol will be presented by treatment arm.

4.4.2 Protocol Deviations

The following protocol deviations, which impact the statistical analyses, will result in exclusion from the Per-Protocol Set (PP):

- Entering the trial when the eligibility criteria should have prevented trial entry
- Discontinuation of intervention prior to 52 weeks
- Use of single-use catheters in more than 20% of catheterizations in the intervention arm
- A new onset exclusion criterium.

4.5 Analysis Populations

The Enrolled set will include all patients who have provided informed consent and have been included into the study data base.

The intention-to-treat (ITT) analysis set will include all patients randomly assigned to a treatment group, who started with the treatment, regardless of whether they completed the treatment or adhered to the protocol.

The modified intention-to-treat (mITT) analysis set will include all patients who were randomly assigned to a treatment group with at least 6 weeks of follow-up data (i.e., those who attended the first clinical follow-up visit).

The Per Protocol (PP) Analysis Set will include all randomised patients meeting the study eligibility criteria who completed the 52 weeks follow-up. (i.e., those who attended the final study visit).

5 Outcome Definitions

5.1 General Definitions and Derived Variables

5.1.1 Body Mass Index (BMI)

Body Mass Index (BMI) = Body weight in kilograms divided by the square of the height in meters.

5.1.2 Urine cultures

Urine samples are obtained and cultured at inclusion (week 0) and final visit (week 52). Significant bacteriuria and thereby a positive urine culture was defined by microbial growth of ≥10^3 cfu/mL of one or more bacterial species in a single catheter urine specimen or in a mid-stream voided urine specimen, following the European Urology Guidelines on Urological Infections.

5.1.3 Other calculations

Age (years) = [(date of baseline – date of birth)/365.25]

Time of event = day of event – day of randomization

5.2 Primary Outcome Definition

The primary outcome is the monthly incidence of UTIs during the study period. The definition of a UTI used for this trial is based on the criteria of Woodford et al (1), on the basis of the EAU guidelines on Neurourology (2) and on the basis of the NHG Guidelines for general practitioners (3).

An UTI is defined by the following two criteria:

1. An acute onset of one or more of the following symptoms: dysuria/pain during catheterization, hematuria, urinary frequency, urinary urgency, suprapubic pain, flank pain, fever (>38°C), rigors, and delirium. In case of a neurogenic bladder: a change in specific symptoms, like increased urinary incontinence, limb spasm and autonomic dysregulation, could be indicative for a UTI.
2. One of the following positive diagnostic tests: urine culture, dipslide, nitrite test, or urine sediment analysis.

5.3 Secondary Outcomes Definitions

5.3.1 Hospitalisation due to UTI

Hospitalisation due to UTI is defined as an admission to a hospital for the management and treatment of a UTI (see primary outcome definition). This occurs when the infection is severe enough to require inpatient care, either due to complications or the inability to manage symptoms adequately in an outpatient setting.

5.3.2 Bacteraemic UTI/ urosepsis

Bacteraemic UTI refers to a UTI associated with bacteraemia, meaning the presence of bacteria in the bloodstream that has originated from the urinary tract. The diagnosis of a bacteraemic UTI is confirmed by blood cultures showing a pathogen typically associated with UTI, alongside clinical symptoms of a UTI. Urine cultures may also provide supportive evidence. The bacteraemia must be determined to have originated from the urinary tract, based on clinical judgment and/or laboratory investigations.

5.3.3 Urethral stricture

Urethral damage leading to clinically significant strictures refers to the development of narrowing or scarring of the urethra that results in significant impairment of urine flow or catheterization and requires clinical intervention. Active screening for urethral strictures will not be performed. If a patient report symptoms, diagnostic tests (such as urethroscopy or urethrography) will be performed to confirm the presence of a urethral stricture.

5.3.4 Kidney/bladder stone formation

Kidney and bladder stone formation is defined as the formation of stones in the kidneys or bladder that occur during the study period and are detected through diagnostic imaging (or cystoscopy) based on symptoms reported by the patient. Active screening for kidney/bladder stones will not be performed.

5.3.5 Episode of macroscopic hematuria

An episode of macroscopic hematuria refers to any occurrence of visible blood in the urine during the study period. Each distinct occurrence (even if it recurs after a period of absence) will be counted as a separate episode.

5.3.6 Patient satisfaction

Patient Satisfaction in this study is defined as the patient's overall satisfaction with their treatment experience, specifically regarding the use of intermittent catheters. Patient satisfaction is measured using the following PROMs:

1) ISCQ (Intermittent Self-Catheterization Satisfaction Questionnaire):
    - Purpose: Measures satisfaction with the process of intermittent self-catheterization.
    - Scoring: The ISCQ consists of multiple items rated on a 5-point Likert scale (1 = Strongly Agree, 2 = Agree, 3 = Neutral, 4 = Disagree, 5 = Strongly Disagree), where higher scores indicate greater satisfaction. The total score is calculated by averaging or summing the individual item scores.
    - Interpretation: Higher scores reflect greater satisfaction with the catheterization process.

2) InCaSaQ (Intermittent Catheterization Satisfaction Questionnaire):
    - Purpose: Evaluates overall satisfaction with intermittent catheterization, including impact on daily life, convenience, and emotional experience.
    - Scoring: Items are rated on a 5-point Likert scale (1 = Very Satisfied, 2 = Somewhat Satisfied, 3 = Neutral, 4 = Somewhat Dissatisfied, 5 = Very Dissatisfied), with higher scores indicating lower satisfaction. The total score is calculated by averaging or summing individual item responses.
    - Interpretation: Higher scores indicate lower satisfaction, while lower scores represent greater satisfaction with catheterization.

Quality of life (QoL)

QoL in this study is defined as the overall well-being and daily functioning of patients, as assessed using the EQ-5D-5L and SF-Qualiveen questionnaire.

- The EQ-5D-5L is a widely used tool to measure health-related quality of life across five dimensions: mobility**,** self-care**,** usual activities**,** pain/discomfort, and anxiety/depression**.** Each dimension is rated on a 5-level scale, ranging from no problems to extreme problems. The composite results from these five dimensions are used to derive a summary index, as well as a visual analog scale (VAS), which reflects the patient's subjective health status on a scale from 0 (worst possible health) to 100 (best possible health).
- The SF-Qualiveen (short form Qualiveen)
    - Purpose: Assesses the health related QoL for urinary disorders in patients.
    - Scoring: Items 1,2,5,6,7,8 use the following response scale:
        - 0 = not at all
        - 1 = Slightly
        - 2 = Moderately
        - 3 = Quite a bit
        - 4 = Extremely

Items 3,4 use the following response scale

- 4 = Never
- 3 = Rarely
- 2 = From time to time
- 1 = Often
- 0 = Always

    - Interpretation: A lower score indicates a greater QoL.

5.3.7 Cost-effectiveness

The cost-effectiveness analysis (CEA) of reusable catheters versus single-use catheters will evaluate the incremental costs and incremental health effects of using reusable catheters, with health effects expressed in quality-adjusted life-years (QALYs). This analysis will follow the Dutch health economic guidelines (4) and be conducted by the Institute for Medical Technology Assessment (iMTA) at Erasmus University, Rotterdam. As such the societal perspective will be adopted, meaning that all costs and effects will be included in the analysis, regardless to whom they accrue. The time horizon

of the cost-effectiveness study will be equal to the timeframe of the clinical trial. Uncertainty concerning the incremental cost-effectiveness ratios, QALYs and costs will be assessed using bootstrapping, and this uncertainty will be presented graphically with the CE-acceptability curve. Data on medical healthcare utilisation (ie, volumes) will be collected both through hospital records and by means of the iMTA Medical Consumption Questionnaire (5). Data on productivity losses will be collected by means of the iMTA Productivity Costs Questionnaire (6). We will use a willingness to pay (WTP) threshold of €20 000/QALY, based on the reference value for cost-effectiveness determined by the National Healthcare Institute of The Netherlands (4). A study on health-economic burden of urinary-catheter-associated infection in England used a similar WTP threshold of £20 000/QALY based on the National Institute for Health and Care Excellence guidelines (7,8). Cost-effectiveness analysis will be presented in separate manuscript.

5.3.8 Patient opinion

Patient opinion regarding healthcare costs and environmental burden in the context of CISC refers to patients' perceptions and attitudes towards the financial and environmental impacts of using single-use versus reusable catheters. This outcome is assessed using two statement-based questions, each rated on a Likert scale from 1 to 5 (where 1 = fully agree, 5 = fully disagree).

5.3.9 Changes in urine cultures over time

Changes in urine cultures over time refers to the evaluation of the bacterial composition of urine samples collected at week 0 (baseline) and week 52 (final visit). This outcome will focus on identifying any changes in bacterial species present in the urine.

Overview of Outcomes

Level	Outcome	Timeframe	Type
Primary	UTIs per month	During trial	Continuous
Secondary	UTI during follow-up	During trial	Dichotomous
	Hospitalisation due to UTI	During trial	Dichotomous
	Bacteraemic UTI/ urosepsis	During trial	Dichotomous
	Urethral stricture	During trial	Dichotomous
	Bladder/kidney stone formation	During trial	Dichotomous
	Epididymo-orchitis	During trial	Dichotomous
	Prostatitis	During trial	Dichotomous

Episodes of macroscopic hematuria	During trial	Dichotomous
Hospitalisation, not study linked	During trial	Dichotomous
Urethral irritation	During trial	Dichotomous
Pyelonefritis	During trial	Dichotomous
ISCQ-score (patient satisfaction)	During trial (4 time points)	Continuous
InCaSa-score (patient satisfaction)	During trial (4 time points)	Continuous
SF-Qualiveen	During trial (4 time points)	Continuous
Index and EQ VAS-score (QoL)	During trial (4 time points)	Continuous
QALYs and total costs	During trial	Continuous
Patient opinion (Likert scale)	Baseline and final visit	Categorical
Changes in urine cultures	Baseline and final visit	Dichotomous
(S)AE	During trial	Categorical/dichotomous

6 Analysis Methods

6.1 Methods for Primary Outcome

6.1.1 Descriptive Statistics

All categorical (binary and ordinal) data will be summarised using frequency counts and percentages of patient incidence. Percentages will be calculated using the study population (FAS); any exceptions to this will be highlighted in the table footnote. The continuous variables will be summarised using number of patients (N), mean, standard deviation (SD), median, 25/75 percentile and range

(minimum/maximum). In general, minimum and maximum will be presented to the same degree of precision as data is recorded, with mean and median having 1 additional place after the decimal and standard deviation having 1 additional places after the decimal. Percentages less than 100 will be displayed to 1 place after the decimal, where space permits.

6.1.2 Primary Analysis

For the primary outcome, the incidence rate in each group is the total number of UTIs divided by the total follow-up time, reported with 95% confidence intervals calculated using bootstrap procedure. The absolute difference between groups will be calculated and reported with a 95% bootstrap confidence interval. Non-inferiority will be concluded if the upper limit of the CI is below the non-inferiority margin of 7%. The incidence rate ratio (IRR) with a 95% CI will be estimated in both arms using a poisson or negative binomial regression model (in case there is overdispersion of the data). All analyses will be conducted using R version 4.2.1 (R Foundation for Statistical Analysis, Vienna, Austria).

6.1.3 Missing Data

For the primary analysis missing data will not be imputed.

6.1.4 Methods (S)AE Primary Outcomes

6.1.4.1 Descriptive Statistics

The frequency (n) and percentage (%) of subjects with any (s)AEs will be summarised by treatment group. SAEs will be detailed and presented in a separate table.

6.1.4.2 Missing Data

Missing data on (S)AE will not be imputed.

6.2 Methods for secondary outcomes

6.2.1 Methods for outcomes of questionnaires (ISCQ, InCaSaQ, EQ-5D-5L, SF-Qualiveen, PGI-I).

6.2.1.1 Descriptive Statistics

Continuous variables will be described using means and standard deviations. Categorical variables will be described using frequency (n) and percentages (%).

Linear mixed models (LMMs) will be used used to assess longitudinal trajectories of the PROs between the two treatment arms. The models will include fixed effects for treatment group, time, and the interaction between time and treatment group. Since all questionnaires are collected at the same follow-up scheme, time will be used as a categorial variable (9). To account for within-subject correlation, a random intercept will be included for each patient. Adjustment for confounders is deemed unnecessary, as stratified randomization is applied. Baseline, as well as the scores at 6 weeks, 26 weeks and 52 weeks are included in the model. Longitudinal linear mixed model analyses correct for missing responses at random (9). The interaction between "time x treatment arm" is included in the model as interaction to assess whether the treatment arm had different effects on the outcomes of the questionnaires at different points in time (9).
A post hoc subgroup analysis is conducted to examine outcomes by gender (male vs. female), age

(<65 vs. ≥65 years), and the ethology of lower urinary tract symptoms (LUTS) (neurogenic vs. non-neurogenic origin), using linear mixed-effects models. The model will include fixed effects for treatment arm, gender, age group, and LUTS ethology, as well as interaction terms between the treatment arm and each subgroup variable. Holm's method for multiple comparisons correction will be applied to adjust p-values. The age cut-off is determined based on the median age of the study population.
To facilitate interpretation of changes in the questionnaire scores, effect sizes will be calculated with the use of Cohen's D test. An effect size of 0.3 or less is considered small, more than 0.3 to 0.8 moderate, and more than 0.8 large (10).

6.2.1.2 Missing Data

Linear mixed models correct for missing responses at random.

6.2.2 Methods for the outcome of Patient opinion questionnaire

6.2.2.1 Descriptive Statistics

The total number of responses per answer will be calculated and presented in a table.

6.2.2.2 Missing Data

Missing data will not be imputed.

6.2.3 Methods for changes in urine culture

6.2.3.1 Descriptive Statistics

The most common bacteria will be showed in a table for both baseline and end of study.

6.2.3.2 Missing Data

Missing data will not be imputed.

6.3 Sample size

The number of studies that have investigated the effects of single use and disposable catheters is limited. Our initial sample size calculation was based on data from a Cochrane review by Prieto et al. (2015), which was later found to contain inaccuracies in data selection and extraction (10). Specifically, six of the eight trials included in the review did not contain consistent or accurate data compared to their originally published versions. Unfortunately, these erroneous data were used as the basis for our initial sample size calculation (n=456). We were therefore required to perform a new sample size calculation based on the revised 2021 Cochrane review with corrected data (11). The revised Cochrane review included ten studies comparing single-use catheters to reusable catheters. To prevent confounding effects related to insertion techniques (clean vs. sterile), six of these studies employed the same insertions method in both study arms. Among these, three studies provided usable data regarding the number of sUTI events in each group. For single use 18 events out of 68 were observed, for reusable 20 events out of 69. This leads to the proportions of 0.26 and 0.29. Further we applied a power of 0.80 and a one-sided alpha of 0.025 (it is customary to adjust one-sided alphas to the half of 0.05). In our previous calculation, the non-inferiority margin had been determined based on 50% of the mean proportions, following the recommendation of Althunian et al. (12) However, with the corrected data, this would have resulted in an increase in the

noninferiority margin to 14%. As this was deemed inappropriate, we opted to maintain the margin at 11%. The sample size is then calculated with: $n=((Z(1-a)+Z(1-ß))^2 [ps (1-ps)+pe (1-pe)])/((ps-pe-d)^2)$, the formula developed by Blackwelder et al in 1982 (13), leading to 154 effective cases in each group. Anticipating a dropout of 20% (14), this must be divided by 80% and rounded upwards. This results in 2 times 193 participants, a total of 386. Because the lack of comparable non-inferiority designed trials with the same primary outcome measurement (sUTI) it is chosen to look at non-inferiority trials with a primary outcome measurement of (treatment of) sUTI. All these trials handled a non-inferiority marge of 10% (15-19), and two trials even 15% (20,21). The head researchers and clinicians of the departments of urology and medical microbiology agreed on the 11% marge to be clinical acceptable. In addition, we encountered the unique challenge of conducting a study with a significantly extended follow-up period of one year, in contrast to the previous studies used in the sample size calculation above, with a shorter follow-up of 2-4 months. Recognizing the impact of time on the occurrence of urinary tract infections (UTIs) and the need to account for this extended observation period, we also performed a sample size calculation that considered the monthly UTI rates observed by Vapnek et al. (22) his calculation factored in not only the proportion of UTIs but also the temporal aspect, recognizing that the incidence of UTIs may vary over time. Vapnek et al. found that single-use catheters had an average of 0.13 UTIs per month (SD 0.18) and multiple-use catheters had an average of 0.14 UTIs per month (SD 0.21) per patient (23). We applied the same power of 0.80 and an one-sided alpha of 0.025. A non-inferiority margin of 7% has been determined based on 50% of the means following the recommendation of Althunian et al. (24) We used the most conservative standard deviation of 0.21 and the sample size calculation was performed using the formula developed by Julious: $n = f(\alpha, \beta) \times 2 \times \sigma^2 / d^2$ (245. This calculation led to a requirement of 152 effective cases in each group. Anticipating a dropout rate of 20%, this figure was divided by 0.80, resulting in a total sample size of 382 participants (191 in each group). Since the sample size of 382 is almost equal to our sample size that does not correct for time (n=386), we will include 386 participants to account for both methods.

Sample size: 386 patients.

7 Safety Analyses

General safety evaluations will be based on the incidence and type of AEs. Safety variables will be tabulated and presented for all patients in the safety set. Safety analysis will be presented in the primary outcomes manuscript.

7.1 Adverse Events

Adverse events are defined as any undesirable experience occurring to a subject during the study, whether or not considered related to the investigational product. All adverse events reported spontaneously by the subject or observed by the investigator or his staff will be recorded.

The number (%) of subjects with any AEs will be summarised by treatment group. SAEs will be detailed and presented in a separate table. Adverse events will be presented in the primary outcomes manuscript.

7.2 Clinical Laboratory Parameters

Not applicable

7.3 Vital Signs

Not applicable

8 Statistical Software

All statistical analyses will be performed using R (R Core Team, 2023. R: A language and environment for statistical computing, version 4.4.1. R Foundation for Statistical Computing, Vienna, Austria. URL: https://www.R-project.org).

9 References

9.1 Literature References

1. Woodford HJ, George J. Diagnosis and management of urinary tract infection in hospitalized older people. J Am Geriatr Soc. 2009;57(1):107-14.
2. Groen J, Pannek J, Castro Diaz D, Del Popolo G, Gross T, Hamid R, et al. Summary of European Association of Urology (EAU) Guidelines on Neuro-Urology. Eur Urol. 2016;69(2):324-33.
3. Chan JL, Cooney TE, Schober JM. Adequacy of sanitization and storage of catheters for intermittent use after washing and microwave sterilization. J Urol. 2009;182(4 Suppl):2085-9.
4. Zwaap J, Knies S, van der Meijden C. Costs-effectiveness in practice 2015.
5. H-vRL BC, Koopmanschap M, Krol M, et al. Manual iMTA medical cost questionnaire (iMCQ) [in Dutch]. Handleiding iMTA medical cost questionnaire (iMCQ), 2013.
6. Bouwmans C, Krol M, Severens H, et al. The iMTA productivity cost questionnaire: a standardized instrument for measuring and Valuing health-related productivity losses. Value Health 2015;18:753-8.doi:10.1016/j.jval.2015.05.009pmid:http://www.ncbi.nlm.nih.gov/pubmed/26409601
7. Smith DRM, Pouwels KB, Hopkins S, et al. Epidemiology and health-economic burden of urinary-catheter-associated infection in English NHS hospitals: a probabilistic modelling study. J Hosp Infect 2019;103:44-54.doi:10.1016/j.jhin.2019.04.010pmid:http://www.ncbi.nlm.nih.gov/pubmed/31047934
8. NICE. Developing NICE guidelines: the manual. United Kingdom, 2014.
9. Twisk JWR. Applied Longitudinal Data Analysis for Epidemiology: A Practical Guide. 2 ed. Cambridge: Cambridge University Press; 2013.
10. Cohen J. Statistical Power Analysis for the Behavioral Sciences. Rev. ed. ed. New York: Lawrence Erlbaum Associates; 1988.
11. Prieto JA, Murphy C, Moore KN, Fader MJ. Intermittent catheterisation for long-term bladder management (abridged cochrane review). Neurourol Urodyn. 2015;34(7):648-53.
12. Vahr S, Cobussen-Boekhorst H, Eikenboom J, Geng V, Holroyd S, Lester M, et al. EAUN Evidence-based Guideline for: Best Practice in Urological Health Care - Catheterisation -

Urethral intermittent in adults. http://wwwuroweborg/nurses/nursingguidelines/. March 2013.

13. Richtlijn Blaaskatheters - Langdurige blaaskatheterisatie bij patienten met complexe multimorbiditeit. Verenso, versie april 2011.
14. Blackwelder WC. "Proving the null hypothesis" in clinical trials. Control Clin Trials. 1982;3(4):345-53.
15. Cardenas DD, Moore KN, Dannels-McClure A, Scelza WM, Graves DE, Brooks M, et al. Intermittent catheterization with a hydrophilic-coated catheter delays urinary tract infections in acute spinal cord injury: a prospective, randomized, multicenter trial. Pm R. 2011;3(5):408-17
16. van Nieuwkoop C, van't Wout JW, Assendelft WJ, Elzevier HW, Leyten EM, Koster T, et al. Treatment duration of febrile urinary tract infection (FUTIRST trial): a randomized placebo-controlled multicenter trial comparing short (7 days) antibiotic treatment with conventional treatment (14 days). BMC Infect Dis. 2009;9:131.
17. van Nieuwkoop C, van der Starre WE, Stalenhoef JE, van Aartrijk AM, van der Reijden TJ, Vollaard AM, et al. Treatment duration of febrile urinary tract infection: a pragmatic randomized, double-blind, placebo-controlled non-inferiority trial in men and women. BMC Med. 2017;15(1):70.
18. Wagenlehner FM, Umeh O, Steenbergen J, Yuan G, Darouiche RO. Ceftolozane-tazobactam compared with levofloxacin in the treatment of complicated urinarytract infections, including pyelonephritis: a randomised, double-blind, phase 3 trial (ASPECTcUTI). Lancet. 2015;385(9981):1949-56
19. Vik I, Bollestad M, Grude N, Baerheim A, Damsgaard E, Neumark T, et al. Ibuprofen versus pivmecillinam for uncomplicated urinary tract infection in women-A doubleblind, randomized non-inferiority trial. PLoS Med. 2018;15(5):e1002569
20. Ten Doesschate T, van Mens SP, van Nieuwkoop C, Geerlings SE, Hoepelman AIM, Bonten MJM. Oral fosfomycin versus ciprofloxacin in women with E.coli febrile urinary tract infection, a double-blind placebo-controlled randomized controlled noninferiority trial (FORECAST). BMC Infect Dis. 2018;18(1):626.
21. Wagenlehner FM, Abramov-Sommariva D, Holler M, Steindl H, Naber KG. Non-Antibiotic Herbal Therapy (BNO 1045) versus Antibiotic Therapy (Fosfomycin Trometamol) for the Treatment of Acute Lower Uncomplicated Urinary Tract Infections in Women: A Double-Blind, Parallel-Group, Randomized, Multicentre, Non-Inferiority Phase III Trial. Urol Int. 2018;101(3):327-36.
22. Ren H, Li X, Ni ZH, Niu JY, Cao B, Xu J, et al. Treatment of complicated urinary tract infection and acute pyelonephritis by short-course intravenous levofloxacin (750 mg/day) or conventional intravenous/oral levofloxacin (500 mg/day): prospective, open-label, randomized, controlled, multicenter, non-inferiority clinical trial. Int Urol Nephrol. 2017;49(3):499-507.
23. Vapnek JM, Maynard FM, Kim J. A prospective randomized trial of the LoFric hydrophilic coated catheter versus conventional plastic catheter for clean intermittent catheterization. J Urol. 2003;169(3):994-8
24. Althunian TA, de Boer A, Groenwold RHH, Klungel OH. Defining the noninferiority margin and analysing noninferiority: An overview. Br J Clin Pharmacol. 2017;83(8):1636-42.

25. Julious SA. Sample sizes for clinical trials with normal data. Stat Med. 2004;23(12):1921-86.
