# Supplementary material for: Single-Use vs Reusable Catheters for Intermittent Catheterization in Patients With Urinary Retention: The COMPARE Randomized Clinical Trial
Source: JAMA Netw Open. 2026 Jun 30;9(6):e2620871. doi: 10.1001/jamanetworkopen.2026.20871 (PMC13320647; doi:10.1001/jamanetworkopen.2026.20871)
Supplement: Supplement 2. — eTable 1. Summary of Analysis Populations eTable 2. Unadjusted Complier Average Causal Effect (CACE) Estimates for the Modified Intention-to-Treat and Intention-to-Treat Populations [file jamanetwopen-e2620871-s002.pdf]

Supplemental Online Content

van Veen FEE, Christiaans CHH, Berendsen SA, et al. Single-use vs reusable catheters for intermittent catheterization in patients with urinary retention: the COMPARE randomized clinical trial. *JAMA Netw Open*. 2026;9(6):e2620871.
doi:10.1001/jamanetworkopen.2026.20871

eTable 1. Summary of Analysis Populations

eTable 2. Unadjusted Complier Average Causal Effect (CACE) Estimates for the Modified Intention-to-Treat and Intention-to-Treat Populations

This supplemental material has been provided by the authors to give readers additional information about their work.

© 2026 van Veen FEE et al. *JAMA Network Open*.

eTable 1. Summary of Analysis Populations

	ITT		Modified ITT		Per-protocol	
	Single-use	Reusable	Single-use	Reusable	Single-use	Reusable
Number analysed	192 (99.5%)	185 (96.4%)	191 (99.0%)	134 (69.9%)	177 (91.7%)	113 (58.5%)
Age, mean (SD), y	61.1 (15.3)	61.7 (16.5)	61.1 (15.3)	62.3 (15.2)	61.4 (15.2)	62.2 (15.5)
BMI, mean (SD), kg/m^2	25.6 (4.0)	26.1 (4.6)	25.7 (4.0)	25.8 (4.3)	25.5 (4.0)	25.6 (4.1)
Sex						
Male	121 (63.0)	118 (64.0)	120 (63.0)	80 (60.0)	112 (63.0)	66 (58.0)
Female	71 (37.0)	67 (36.0)	71 (37.0)	54 (40.0)	65 (37.0)	47 (42.0)
NLUTD	69 (36.0)	68 (37.0)	68 (36.0)	46 (34.0)	65 (37.0)	42 (37.0)
Spontaneous miction	106 (55.0)	85 (46.0)	106 (55.0)	68 (51.0)	96 (54.0)	58 (51.0)
CIC experience						
< 0.5 years	22 (12.0)	19 (10.2)	22 (12.0)	13 (9.8)	18 (10.0)	11 (9.8)
0.5 - 1 years	19 (10.0)	31 (17.0)	19 (10.0)	22 (17.0)	17 (9.7)	18 (16.0)
1 - 3 years	66 (35.0)	54 (29.0)	66 (35.0)	39 (29.0)	63 (36.0)	33 (29.0)
≥ 3 years	82 (43.0)	80 (43.0)	81 (43.0)	59 (44.0)	78 (44.0)	50 (45.0)
Unknown	3	1	3	1	1	1
CIC frequency						
2-4x/day	73 (38.0)	58 (28.0)	73 (38.0)	48 (36.0)	65 (37.0)	41 (37.0)
5-6x/day	80 (42.0)	90 (48.0)	80 (42.0)	63 (48.0)	77 (44.0)	53 (48.0)
≥ 7x/day	36 (19.0)	35 (10.0)	35 (18.0)	21 (16.0)	32 (18.0)	17 (15.0)
Unknown	3	2	3	2	3	2
Self-reported UTIs in previous 6 months before trial entry						
0x	133 (69.0)	129 (70.0)	132 (69.0)	102 (76.0)	125 (71.0)	86 (76.0)
1-2x	51 (27.0)	45 (24.0)	51 (27.0)	27 (20.0)	45 (25.0)	22 (19.0)
≥ 3x	8 (4.1)	11 (5.9)	8 (4.1)	5 (3.6)	7 (4.0)	5 (4.5)

Abbreviations: BMI, body mass index; CIC, clean intermittent catheterization; ITT, intention-to-treat; NLUTD, neurogenic lower urinary tract dysfunction; SD, standard deviation; UTI, urinary tract infection.

© 2026 van Veen FEE et al. *JAMA Network Open*.

eTable 2. Unadjusted Complier Average Causal Effect (CACE) Estimates for the Modified Intention-to-Treat and Intention-to-Treat Populations

Analysis	Estimate (UTIs/patient-month)	95% CI	p-value	F-statistic	Wu-Hausman p-value
CACE unadjusted ITT	0.002	-0.067 to 0.071	0.95	121.7	0.41
CACE unadjusted mITT	0.016	-0.126 to 1.158	0.83	35.3	0.26

Abbreviations: CACE, Complier Average Causal Effect; CI, confidence interval; ITT = intention-to-treat; mITT, modified ITT. F-statistic refers to the weak instruments test; value > 10 indicate a strong instrument. Wu-Hausman p-value tests whether non-compliance biased the primary analysis; p>0.05 indicates meaningful bias.

© 2026 van Veen FEE et al. *JAMA Network Open*.
