# Supplementary material for: Single-Use vs Reusable Catheters for Intermittent Catheterization in Patients With Urinary Retention: The COMPARE Randomized Clinical Trial
Source: JAMA Netw Open. 2026 Jun 30;9(6):e2620871. doi: 10.1001/jamanetworkopen.2026.20871 (PMC13320647; doi:10.1001/jamanetworkopen.2026.20871)
Supplement: Supplement 3. — Data Sharing Statement [file jamanetwopen-e2620871-s003.pdf]

Data Sharing Statement

van Veen. Single-Use vs Reusable Catheters for Intermittent Catheterization in Patients With Urinary Retention. *JAMA Netw Open*. Published June 30, 2026.
doi:10.1001/jamanetworkopen.2026.20871

Data
Additional Information: National Trial Register (NTR): NL8296
Data available: Yes
Data types: Deidentified participant data
How to access data: The de-identified participant data, statistical code, and any other study materials (including data dictionary) will be made available upon reasonable request. Requests can be directed to F.E.E. van Veen at f.vanveen@erasmusmc.nl. Access will be provided following approval of a research proposal and a signed data use agreement.
When available: With publication

Supporting Documents
Document types: Informed consent form, Statistical/analytic code
How to access documents: The de-identified participant data, statistical code, and any other study materials (including data dictionary) will be made available upon reasonable request. Requests can be directed to F.E.E. van Veen at f.vanveen@erasmusmc.nl. Access will be provided following approval of a research proposal and a signed data use agreement.
When available: With publication

Additional Information
Who can access the data: The de-identified participant data, statistical code, and any other study materials (including data dictionary) will be made available upon reasonable request. Requests can be directed to F.E.E. van Veen at f.vanveen@erasmusmc.nl. Access will be provided following approval of a research proposal and a signed data use agreement.
Types of analyses: The de-identified participant data, statistical code, and any other study materials (including data dictionary) will be made available upon reasonable request. Requests can be directed to F.E.E. van Veen at f.vanveen@erasmusmc.nl. Access will be provided following approval of a research proposal and a signed data use agreement.
Mechanisms of data availability: The de-identified participant data, statistical code, and any other study materials (including data dictionary) will be made available upon reasonable request. Requests can be directed to F.E.E. van Veen at f.vanveen@erasmusmc.nl. Access will be provided following approval of a research proposal and a signed data use agreement.
